# A skin secretion metabolome analysis of the Greek Dodecanese Lycian salamanders: Preliminary evidence of dietary alkaloid sequestration in urodeles

**DOI:** 10.1371/journal.pone.0300278

**Published:** 2024-08-29

**Authors:** Karolos Eleftherakos, Roza Maria Polymeni, Eleni V. Mikropoulou, Konstantina Vougogiannopoulou, Christos Georgiadis, Eleftherios A. Petrakis, Leandros A. Skaltsounis, Maria Halabalaki

**Affiliations:** 1 Section of Zoology–Marine Biology, Department of Biology, National and Kapodistrian University of Athens, Athens, Greece; 2 Division of Pharmacognosy and Natural Products Chemistry, Department of Pharmacy, National and Kapodistrian University of Athens, Athens, Greece; Institute for Biological Research, University of Belgrade, SERBIA

## Abstract

*Lyciasalamandra* species, like most amphibians, secrete a wide array of compounds from their granular and mucous skin glands, including the internally synthesized samandarine alkaloids, making their skin a complex organ performing a variety of functions. *Lyciasalamandra helverseni* and *L*. *luschani basoglui* are insular endemics of the Dodecanese islands of SE Greece, bearing distinct isolated populations, with well-documented phylogenetic profiles. Here, we employ a metabolomics approach, utilizing UPLC–ESI-HRMS/MS data of the skin secretions sampled from a number of specimens found in the islands of Karpathos, Kasos and Kastellorizo, in an effort to reveal aspects of their chemistry and diversity across populations. The results indicated statistically significant variation between all taxa examined, based on various secreted compounds. The underlying factors of variation highlighted by the multivariate analysis were differences in samandarine and other alkaloid content as well as in animal size. Metabolite annotation, based on dereplication tools and most importantly HRMS and HRMS/MS spectra, yielded a number of known samandarine alkaloids, reported for the first time in the currently studied *Lyciasalamandra* species. We also present documentation for novel members of the samandarine alkaloid family, as well as preliminary evidence for a possible dietary alkaloid sequestration. This work can set the basis for further research of this often-neglected endemic species of the Salamandridae, as well as the structural investigation of the samandarine alkaloid group.

## Introduction

**Fig 1:**
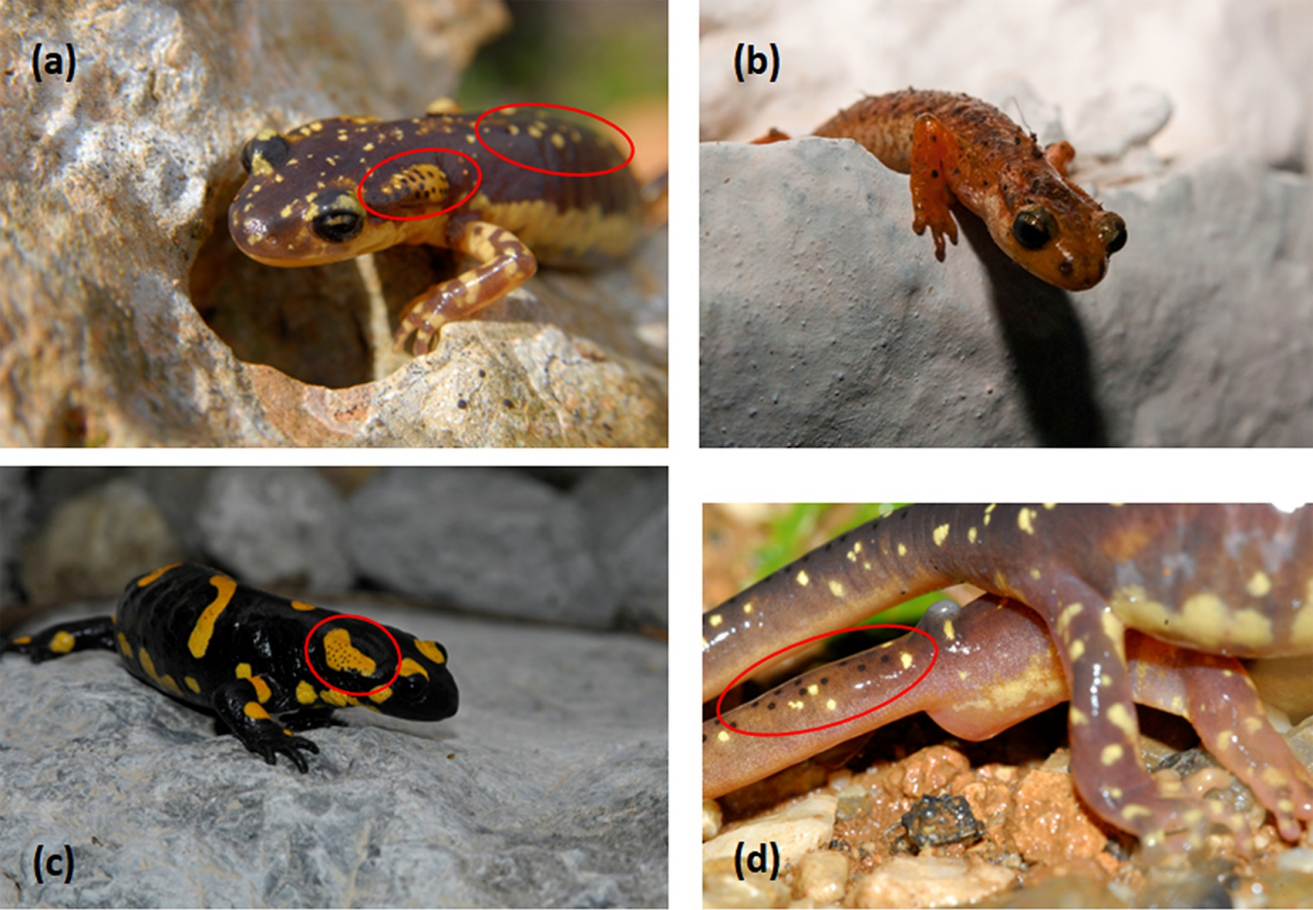
Representative specimens of the salamander species used in the present study. Red circles indicate cutaneous glands (parotoids, dorsal and tail). (a) *Lyciasalamandra helverseni* (Karpathos salamander), (b) *Lyciasalamandra luschani basoglui* (Luschan’s salamander), (c) *Salamandra salamandra* (fire salamander), (d) *Lyciasalamandra helverseni* in amplexus. Notice the hedonic gland in the dorsal side of the base of the tail.

Lycian salamanders (*Lyciasalamandra* spp.) are species of a polytypic salamander genus (family: Salamandridae), endemic to the Southeastern Aegean Greek islands of Karpathos, Kasos and Saria, as well as Kastellorizo and the adjacent Southwestern Turkish coast (S1 Fig in S1 File). The genus comprises seven species which are all comparatively small, resembling one another, and they are basically distinguished macroscopically by subtle differences in coloration and morphometric parameters [1–3]. The basic shape resembles that of the fire salamander (*Salamandra* sp.), though much smaller and gracile, with the same dorsal surface skin glands, but lacking the striking aposematic coloration (Fig 1). Also, male species of Lycian salamanders possess a hedonic gland in the dorsal side of the base of the tail (a homologous structure to the protuberance of *Mertensiella caucasica*) used for mating (Fig 1) [3].

*Lyciasalamandra helverseni* (LH) and *Lyciasalamandra luschani basoglui* (LLB) are species of restricted distribution (S1 Fig in S1 File). The former is isolated to Karpathos, Kasos and Saria islands, the latter native to Kastellorizo island and the adjacent mainland. Both are considered vulnerable (VU) according to the IUCN red list. A network of various, more or less isolated populations inhabit limestone outcrops, with or without tall vegetation (pine forests), a fact overstated in LH in Kasos island, where vegetation is vestigial [4].

*Lyciasalamandra* is a comparatively recently described genus [3], previously positioned in the genus *Mertensiella* [5] or suggested to be placed under *Salamandra* [6]. The genus is sister taxon to *Salamandra* [6–9], with estimated time to most recent common ancestor (tmrca) dates ranging from 11 million years ago (mya) [6], 9.29 (95% CI: 6.12–12.8 mya) [10], up to 25.1 mya [11] depending on calibration dates and methodology. It includes seven species [1–3, 11], with various subspecies. Its phylogeny is resolved mostly by means of molecular marker diversification, namely allozyme frequencies, mtDNA haplotypes, nuclear DNA sequences as well as morphometrics [3, 4, 6, 8, 9, 12]. Their life cycle is completely independent of water bodies, which leads to a very much confined activity period, depended instead on a relative humidity threshold of about 60% [13] for any activity over ground; *Lyciasalamandra* species are viviparous and give birth to only one or two fully metamorphosed offspring per female per year [14]. A recent study concluded that the generally subterranean life mode diminish intra and inter species variation in demographic life history and age-adjusted size traits, while any variation present is explained by differences in above ground activity duration [15].

Amphibian skin in general constitutes a morphologically, biochemically and physiologically complex organ which fulfills a wide range of functions necessary for the organism’s survival. It is involved in respiration, water regulation, anti-predator, anti-microbial and anti-fungal defense, excretion, temperature control, reproduction, etc. [16]. In the biochemical perspective, amphibian granular glands are known to excrete alkaloids, biogenic amines, steroids, anti-microbial peptides (AMPs), and proteins [17–25].

A particular class of metabolites, generally found in the skin of amphibians, are chemical compounds produced by symbiotic bacteria (Proteobacteria, Actinobacteria, Bacteroidetes, Firmicutes, Alphaproteobacteria, Flavobacteriales, Pseudomonadales, Rhizobiales, Firmicetes, Verrucomicrobia, etc.). They develop in the secretions of the mucous glands (mucosome) [26–30] and often have antifungal properties [27, 31], e.g. the mucosal secretion products of *S*. *salamandra mucosa*, which reduce the survival of *B*. *dendrobatidis* and *B*. *salamandrivorans* spores, albeit to a lesser extent than the secretions of the serous glands [32].

As mentioned earlier, the genus *Lyciasalamandra* is sister taxon to the genus *Salamandra*, species of which are well known to synthesize and secrete toxic alkaloids [17, 33, 34]. In salamanders, the secretory product is whitish and sticky [35], and the fire salamander has the ability to spray it directionally at high velocity (>300 cm/s) in response to simulated predator attack. The composition of the secretion includes catalase [36], cholesterol and steroidal alkaloids [37, 38], serotonin and tryptamine [39], hemolytically active peptides [40] as well as other peptides [41] and is secreted from cutaneous granular glands located on the dorsal surface of the body. The greatly enlarged, specialized skin glands are individually encased by connective tissue sheaths and embedded in the epaxial musculature [42]. *Salamandra* species are some of a few amphibians that synthesize their own alkaloids or biogenic amines, along with *Pheudophryne coriacea* (Myobatrachid frogs) [43] and *Melanophryniscus moreirae* (Bufonid frogs) [44]. The two latter also possess the ability of dietary alkaloid sequestration, which refers to the innate ability to isolate and store in exocrine specialized serous glands, alkaloids from dietary items. However, sequestration phenomena have not been reported for any salamander species so far.

Similar to fire salamanders, Lycian salamanders also excrete a milky substance when force is applied on the skin glands. The excretion has an intense smell (more so in *L*. *helverseni* than *L*. *l*. *basoglui*, similar to other *Salamandra* species, probably mirroring differences in volatile compounds [45]) and is irritant to the eyes and nose (personal observations). As fire salamanders, they also have the ability to spray the gland contents [42] (personal observations), while only recently it was shown with the aid of GC-MS analysis, that the excretion contains alkaloids [8].

The steroidal alkaloids produced by *Salamandra* and *Lyciasalamandra* species belong to the chemical group of samandarines [34] and so far eleven members have been reported. Despite the fact that the existence of samandarines was discovered in the late 1800’s by E.S Faust [46], and the first basic chemical features were discovered in the 50’s and 60’s by C. Schöpf and G. Habermehl [34, 40], there are only few reports later. Very recently, in 2019 two novel samandarines were identified i.e. *O*-3-hydroxybutanoylsamandarine and samanone by Knepper and co-workers [47]. Nevertheless there have been also recent indications of unknown alkaloids in *Lyciasalamandra* secretions [8].

**Fig 2:**
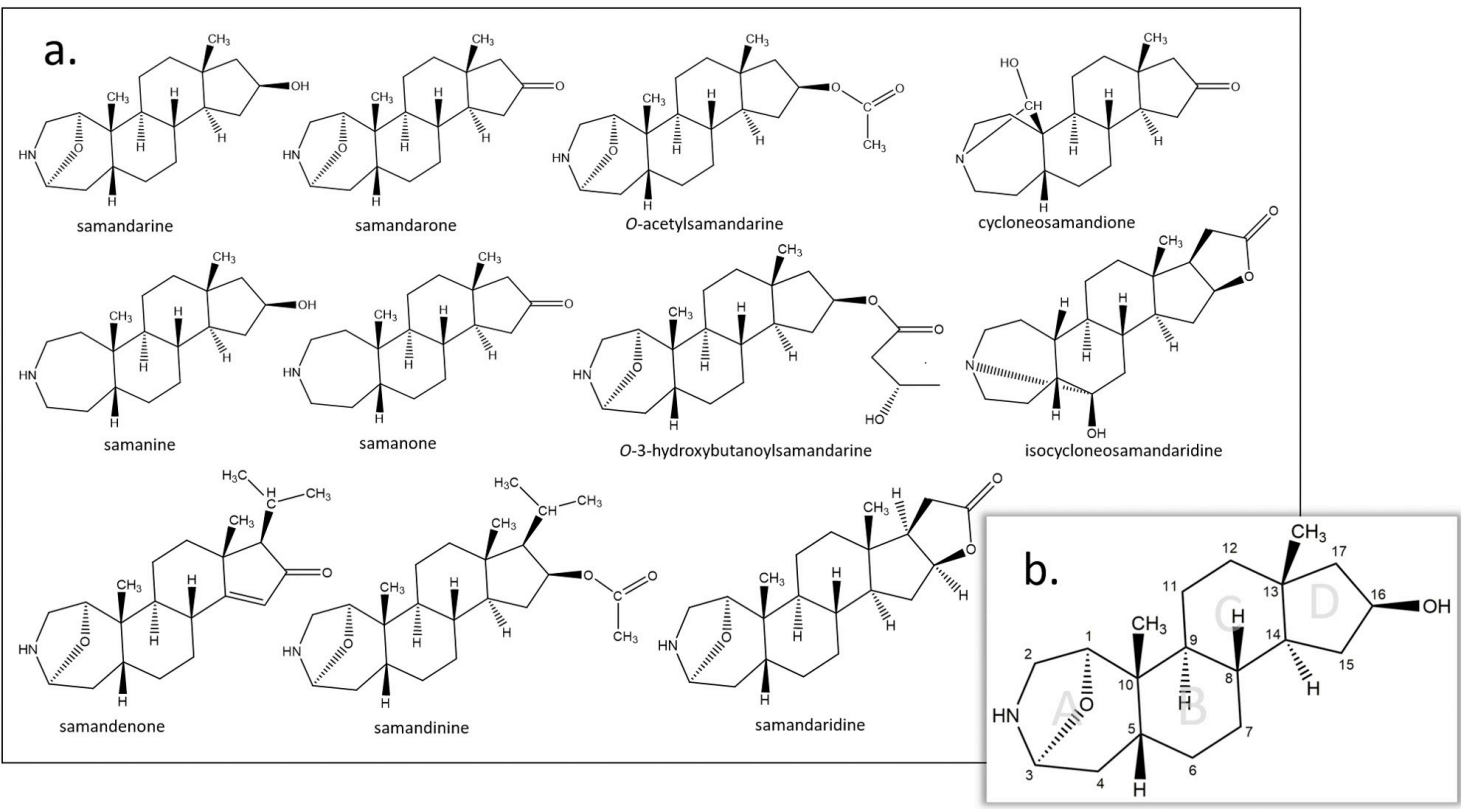
a). The samandarine steroidal alkaloid family. b). Samandarine, with labeled rings and numbered carbon atoms based on Knepper et al [47].

From a chemical point of view, seven samandarines from the reported so far have an oxazolidine system in the A ring (samandarine, samandarone, *O*-acetylsamandarine, samandaridine, samandenone, samandinine, *O*-3-hydroxybutanoylsamandarine); two have a carbinolamine system (cycloneosamandione, isocycloneosamandaridine); and two have neither (samanine, samanone) (Fig 2). The characteristic basic skeleton of the alkaloids is that of a 2a-aza-*A*-homo-5β-steroid and, as exemplified by samandarine, samandarone, and samandaridine, there is usually a 1α,3α-oxide bridge and further oxygenation on C_16_ [48]. As has been described for *S*. *salamandra*, samandarines are formed biosynthetically from cholesterol where the side chain on the D ring is degraded by functionalization with carboxyl groups and sequential decarboxylation reactions performed by enzymes. Thus, the alkaloids with side chain at C_17_ might be considered as intermediates on the way from cholesterol to the alkaloids without side chains [35].

Samandarine alkaloids are highly toxic to mammals, affecting the central nervous system (CNS), and induce death by respiratory paralysis, without damaging the heart. They exhibit toxicity to all higher animals including other amphibians. Lethal dose is 19 mg/kg for frogs, 3.4 mg/kg for mice and 1 mg/kg for rabbits, and even larger mammals like dogs are affected [46, 49–54]. Samandarines (mainly samandarine, samandarone, and samandaridine) also display mild antimicrobial activity, with samandarone being the most potent in this respect [55], though defense against pathogens is mostly attributed to unknown peptides [32].

Recently, it has been suggested that samandarine synthesis is ancestral to Salamandridae [8], thus increasing the number of known amphibian species with alkaloid synthesis capability. All species of the genus *Salamandra*, two species of *Lyciasalamandra* as well as other representatives of Salamandridae, have been found to synthesize members of the steroidal alkaloid group [8, 34, 40, 56], making it obvious that samandarine production has evolved much earlier than previously assumed. So far however, there are no available data regarding the secretions of the two endemic *Lyciasalamandra* species of Greece. Interestingly, it has even been proposed that the diversity of the endogenous steroidal alkaloids of true salamanders, the samandarines, could be partly attributed to microsymbionts of the mucosome [45]. Sphingolipid biosynthesis has also been identified in the *Salamandra* mucosome [26].

It is also worth mentioning, that there are differences found between species regarding the number of different samandarines present [34, 37], the quantity of the secretion the variability of different samandarines between members of the same species, as well as in the same individuals over time [57]. It has been suggested that species of the Salamandridae, plus *Pheudophryne coriacea* [43] and *Melanophryniscus moreirae* [44] are capable of producing these noxious compounds through biosynthesis, nevertheless most amphibian alkaloids are basically accumulated from dietary sources, sequestered in their granular skin glands [58–60]. Currently, there is no systematic investigation whether salamandridae alkaloids are exclusively biosynthesized or are a result of both biosynthesis and sequestration from food items.

**Fig 3:**
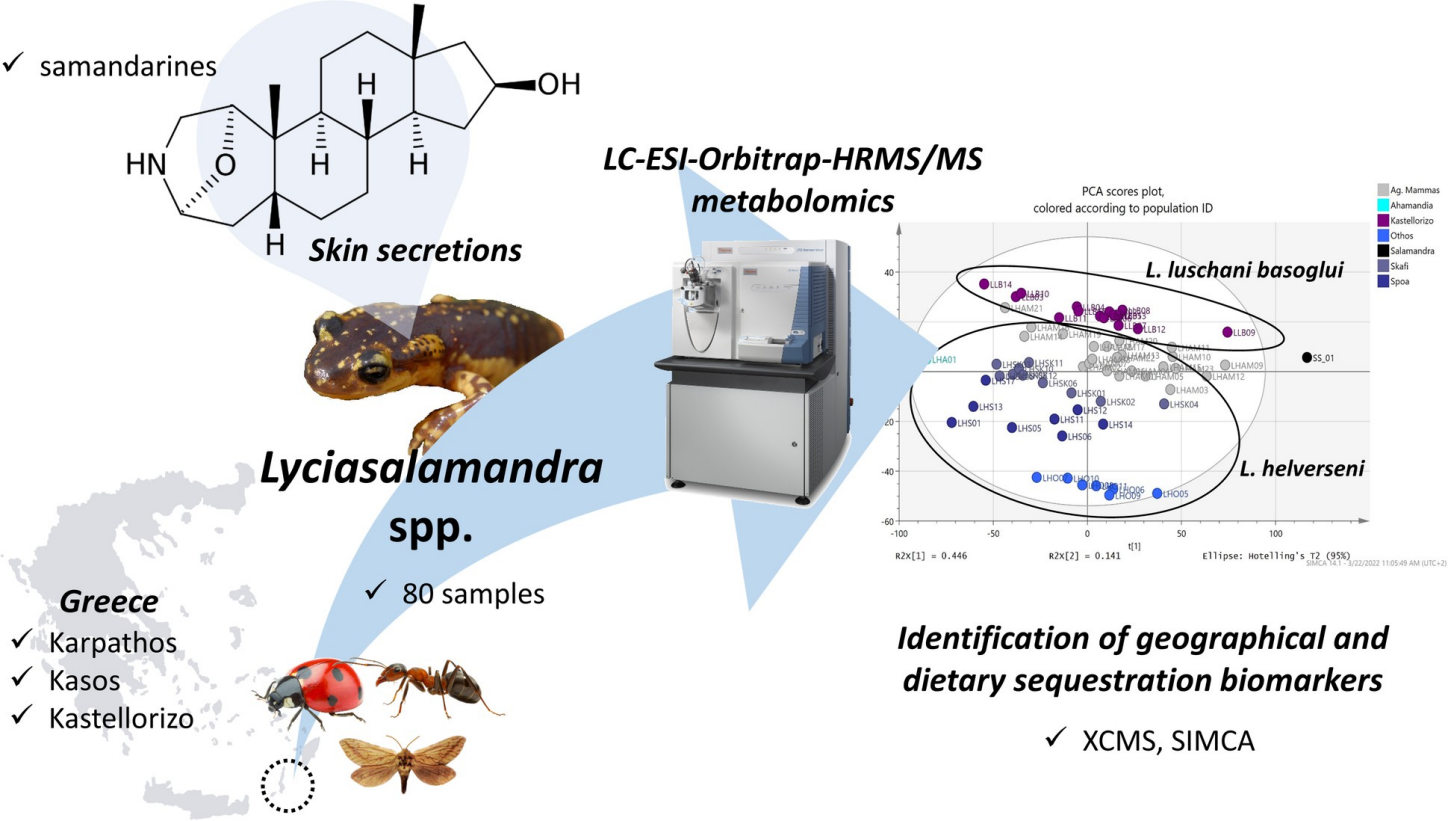
Outlay of the presented study.

Based on the above, in the present study we employed an untargeted metabolomic approach with data generated by LC-HRMS/MS, in order to make full use of any diversification in the skin excretion compound contents of the specimens of LH and LLB in each sampled population, in an effort to uncover underlying geographic subdivision and the reasons behind it (Fig 3). Given the comprehensive phylogeography and distribution of genetic diversity of the two species [4, 11], it is relevant to look for, and compare any patterns of corresponding diversification in the skin secretion metabolome, and to describe and possibly explain any differences between specimens, sexes, population and species regarding it. Furthermore, an attempt to examine possible dietary sequestration aspects and relevant biomarkers was made.

## Materials & methods

### Chemicals

For UPLC-HRMS analysis, acetonitrile (ACN) and formic acid (FA) of LC-MS grade were acquired from Fluka (Buchs, Switzerland). Ultrapure water was obtained from a Milli-Q® purification system (Merck Millipore, Darmstadt, Germany) and also used for the mobile phase. HPLC and LC-MS grade methanol (MeOH) for sample collection and preparation, respectively, was supplied by Fluka. Chloroform (99.8 atom % D) for NMR analysis was purchased from Euriso-Top (Saclay, France).

### Sample collection

A total of 80 secretion samples, belonging to two Lycian salamander species were collected from the islands of Karpathos, Kasos, and Kastellorizo (Table 1). One is exclusively insular (LH) and endemic to Karpathos, Kasos, and Saria islands, and the second (LLB) is found both on mainland Turkish south-west shore as well as on small islands of the S. Aegean area (Kastellorizo, Kekova). In addition, one secretion sample from a *Salamandra salamandra* ssp. *salamandra* specimen collected in mainland Greece (Northern Peloponnese, Chelmos Mountain) was analyzed as reference for the identification of salamander alkaloids. A total of 6 *Lyciasalamandra* populations were sampled. All samples were collected within the limits of protected sites, parts of the Natura 2000 European network of protected areas. In addition, Lycian salamanders sampled herein are listed in the *Annex II of the Directive 92/43/EEC*, as also in the *IUCN Red List of Threatened Species*. No permits were obtained for the samplings, because at the time they were not issued by the Greek Ministry of Environment & Energy. However, the current research was conducted in the context of a Doctoral Dissertation as research supervised by the National and Kapodistrian University of Athens and falls under provision which meets the legal requirements set by the Article 6(2) of Presidential Decree 67/81 which states that National Higher Education Institutions were not required to obtain research permits, but only to communicate the ongoing investigation to the local competent authorities. All secretion samples were collected *in situ*, with all specimens released immediately after collection. No tissue samples nor live specimens were collected, and no animals were harmed during the sampling effort.

**Table 1:** Sampling localities. Geographical subareas, number of samples and specific coordinates are given.

Species	LH					LLB	SS
Locality	Karpathos			Kasos		Kastellorizo	Chelmos mtn
population	Othos	Spoa	Achamandia	Ag. Mammas	Ag. Haralambos Skafis	Kastellorizo	-
N	11	17	1	23	12	16	1
GPS coordinates	35° 32.654’N,	35° 36.168’N,	35° 41.270’N,	35° 23.773’N,	35° 24.436’N,	36° 8.928’N,	37° 59.185’N,
	27° 9.027’E	27° 8.970’E	27° 8.741’E	26° 56.883’E	26° 58.414’E	29° 35.518’E	22° 16.438’E

A *Coccinella septempunctata* specimen was also collected from Attica region, as well as all plant specimens analyzed herein (*Senecio vulgaris*, *Glaucium flavum*, *Papaver rhoeas*, *Fumaria* spp.), in order to obtain HRMS/MS spectra. Suspect analysis for certain compounds was performed and the data were compared with those resulted from *Lyciasalamandra* skin secretion analysis, thus assisting to their annotation. All species are part of the fauna and flora of the islands where secretion samples were collected [61]. Lastly, a bibliographical review of the ant and mite fauna of the islands was performed, so as to explore if typical sources of possible dietary sequestered alkaloids coexist with local *Lyciasalamandra* populations.

### Sample preparation

Skin secretions were obtained on site by means of pressure applied on parotoid, dorsal and tail granular glands, and were adsorbed on thin cellulose paper due to their limited yield. Subsequently, the individual soaked papers were dissolved in HPLC grade MeOH (~30 mL). Salamander secretions were directly injected for UPLC-HRMS/MS analysis. For the NMR analysis, 30 samples were combined, evaporated, and reconstituted in CDCl_3_ solvent. The *Coccinella septempunctata* specimen was prepared and extracted with MeOH using ultrasounds for 2 hours. After centrifugation the supernatant was directly injected to LC-MS. Finally, the plant samples were pulverized and extracted with MeOH using ultrasounds for 2 hours. After centrifugation the supernatant was directly used for analysis.

### UPLC-HRMS/MS and NMR analysis

Samples of all specimens were subjected to UPLC-HRMS/MS analysis, performed using an Acquity H-Class ultra-high performance liquid chromatography (UPLC) system (Waters Corp., Milford, USA) hyphenated to a hybrid LTQ-Orbitrap Discovery XL apparatus (Thermo Scientific, Brehmen, Germany), equipped with an electrospray ionization (ESI) source. Xcalibur 2.0.7 (Thermo Scientific) software was employed for data acquisition and processing. The chromatographic analysis was carried out using a reversed-phase Fortis C_18_ (Fortis Technologies Ltd, Cheshire, UK) column (100 × 2.1 mm, 1.7 μm), heated at 25°C. A gradient method was developed for the chromatographic analysis of samples, with a mobile phase composed of water (solvent A) and acetonitrile (solvent B), both containing 0.1% (*v/v)* FA. The flow rate was 0.4 mL/min and the injection volume was 10 μL. The gradient ratio started with 5% of solvent B for 3 min, then increased linearly to 100% of solvent B at 21 min and was held isocratically until 23 min. Afterwards, the column was stabilized by reversing the gradient to the initial conditions within 2 min and was held stable for another 5 min to a total of 30 min for each run. A pre-run injection of a blank solution was employed for subtracting background.

The mass spectrometer was tuned for standard mass range analysis and data were continually acquired in positive (ESI+) ionization mode, using the full scan range of *m/z* 115–2000 for all samples. The mass resolution was set at 30000, while capillary temperature was 275°C. The tuning of capillary voltage and tube lens was set at 49 and 100 V, respectively. Source voltage was 3.70 kV, while source current was 100 μA. MS^2^ spectra were recorded by selecting data-dependent acquisition, with a collision-induced dissociation (CID) value of 35% and a resolution of 7500. The sheath and auxiliary gas were nitrogen, with the flow rate set at 30 and 10 arbitrary units, respectively.

Nuclear magnetic resonance spectroscopy: nuclear magnetic resonance (NMR) spectra were recorded on a 600 MHz Avance III NMR spectrometer (Bruker Biospin GmbH, Rheinstetten, Germany), operating at ^1^H frequency of 600.11 MHz and ^13^C frequency of 150.89 MHz. 1D and 2D NMR spectra (^1^H, ^13^C, COSY, COSY-LR, HSQC-DEPT, HMBC) were acquired on a Bruker Advance 600 MHz spectrometer using CDCl_3_ as solvent. Chemical shifts (*δ*) are expressed in ppm with reference to the solvent signals (*δ* H 7.26/ *δ* C 77.0). Data acquisition and processing was carried out using TOPSPIN 3.5 (Bruker Biospin GmbH).

### Data processing and chemometrics

Prior to multivariate analysis of UPLC-HRMS/MS data, pre-processing was carried out using the XCMS v1.5 package. After feature detection, using matched filter algorithm [62] an isotope and adducts annotation step followed using the CAMERA package, useful for the subsequent metabolite identification effort. XCMS parameter optimization was performed using the IPO package. All packages were implemented in R/RStudio environment and provided by Bioconductor (http://www.bioconductor.org/). The database resulting from the above procedure contained 7516 entries (*m/z*—Rt). To avoid statistical error due to the variation in the amount of secretion (sampling), all responses in all samples were divided by the ratio of the weight of each sample to the weight of the sample of *Salamandra salamandra* (the heaviest), so that all the ratios range from 0–1 (S2 File). Data filtering, handling of missing values, transformation and scaling steps, as well as multivariate data analysis were conducted using SIMCA 14.1 (MKS Umetrics, Umea, Sweden). To remove heteroscedasticity of data, a logarithmic transformation and a non-linear power (1/4; square root) transformation [63] was used (for PCA and PLS-DA, OPLS-DA respectively). Afterwards, data were pre-treated using Pareto scaling, which reduces the relative importance of large fold changes but keeps the scaled dataset close to the original.

Principal component analysis (PCA) was employed for unsupervised exploratory analysis, with a view to investigate possible patterns among different populations. Partial least squares-discriminant analysis (PLS-DA) and orthogonal projections to latent structures-discriminant analysis (OPLS-DA) were subsequently performed for supervised classification of samples. To investigate the non-randomness of the classification models, permutation tests were performed by calculating a total of 200 models and randomizing the order of Y variable in the corresponding PLS-DA models. Hotelling’s T^2^ and distance to the model (DModX) tests were applied to verify the presence of outliers and to evaluate whether samples fall within the model applicability domain. With a view to select the variables contributing the most to the classification of samples, variable importance in projection (VIP) indices were estimated; 2520 variables with VIP scores larger than 1.0 were finally selected, over 7516 variables originally present for the OPLS-DA. Model quality was described by *R*^*2*^*X* and *Q*^*2*^ values. *R*^*2*^*X* represents the proportion of variance explained by each model, indicating the goodness of fit, while *Q*^*2*^ is calculated by cross-validation and implies the predictive ability of the model. For testing hypothesis, e.g., significant differences in PC score means among biogeographical units, or in known compound mean peak heights per biogeographical unit, a one-way ANOVA was implemented, and regression analysis was used to investigate if and how certain variables (Snout to Vent Length (SVL, S3 File) as an indicator of the animal’s developmental stage, peak heights) affect PC scores.

### Dereplication and identification process

In order to identify known but also not previously reported samandarine alkaloids in the samples, certain dereplication tools were employed. Specifically, chromatographic and spectrometric features i.e., proposed Elemental Composition (EC), Ring Double Bond equivalents (RDBeq.) values, isotopic motifs of pseudomolecular ion, detection and *m/z* measurements accuracy as expressed by *Δ*m (*m/z* theoretical vs experimental) as well as fragmentation patterns in tandem MS spectra were utilized. Proposed ECs were considered only if *Δ*m was < 5ppm. It is important to note that the aforementioned tools were applied in both full scan and HRMS/MS data. Moreover, different available databases such as Metlin, Chemspider, HMBD were used in the identification process as well as thorough examination of literature, while Sirius 5.8.0 [64] was also employed in the dereplication procedure. Finally, 1D and 2D NMR experiments were also performed in selected *Lyciasalamandra*, as well as the *S*. *Salamandra* samples, aiming to increase the identification confidence.

## Results and discussion

### Alkaloid identification in skin secretions of Lycian salamanders

#### Samandarines

**Fig 4:**
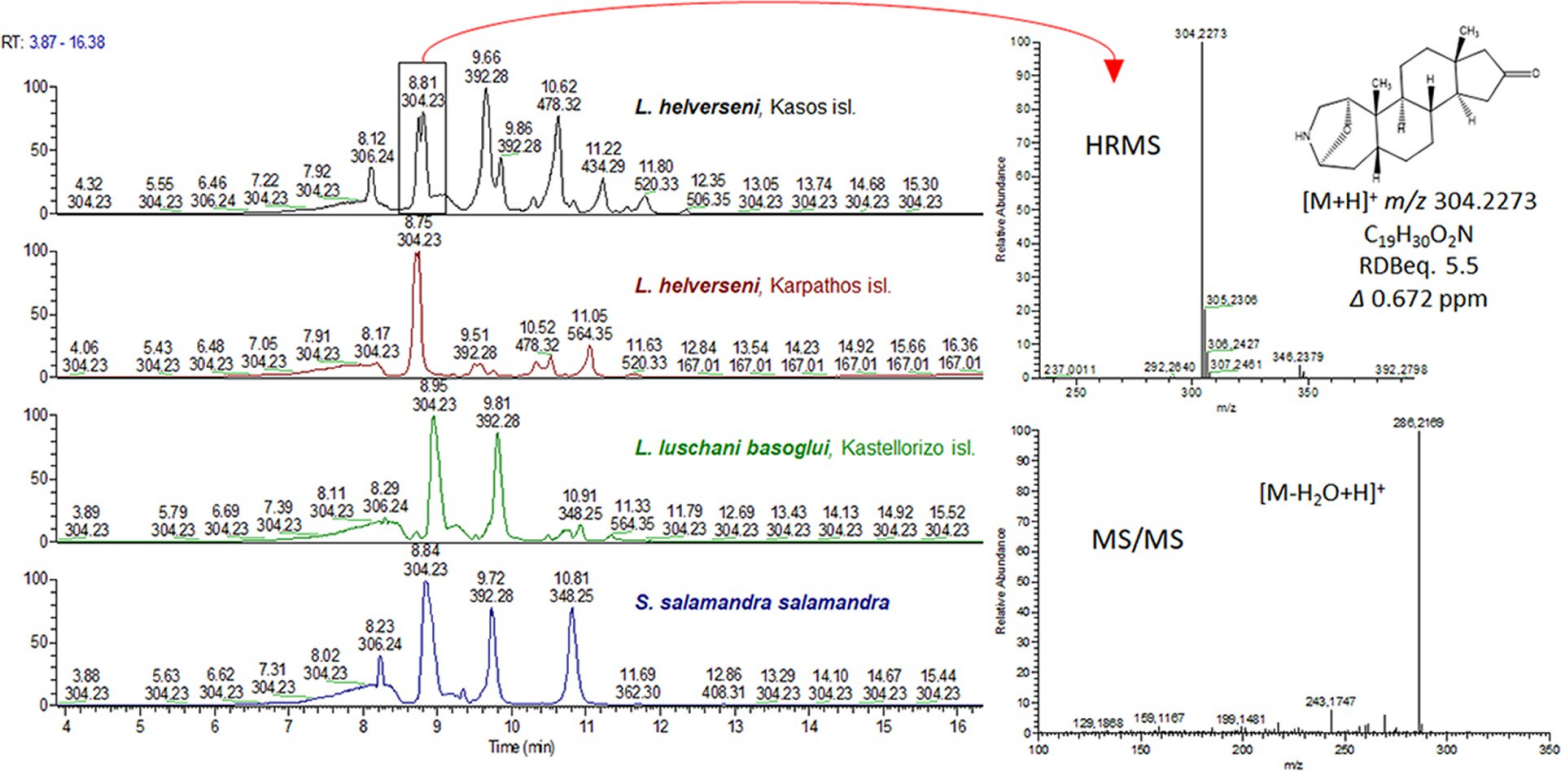
Left: Characteristic LC-HRMS base peak chromatograms of *Lyciasalamandra* specimens from all islands sampled as well as *S*. *salamandra*. Right: indicative HRMS and HRMS/MS spectra of samandarone.

The first step after data acquisition was the detailed examination of the samples’ profiles aiming to identify known samandarines as well as possible new ones exploiting the sensitivity, mass resolution and accuracy of the Orbitrap mass analyzer. Based on dereplication tools and most importantly HRMS and HRMS/MS spectra (Fig 4), a number of known samandarines were identified in the skin secretions of the two *Lyciasalamandra* species for the first time (samandarine, samandarone, *O*-acetylsamandarine, samandaridine, samanine, samanone, *O*-3-hydroxybutanoylsamandarine), and some for the first time in the genus (samandaridine, samanine, samanone, *O*-3-hydroxybutanoylsamandarine). Additionally, a number of unknown compounds were identified as putative novel samandarines (Table 2).

**Table 2:** Tentative identification of samandarines (known and some selected currently unknown) in the skin secretions of *Lyciasalamandra* species by means of UPLC-ESI(+)-HRMS.

Rt (min)	Experimental *m/z* [M+H]^+^	Suggested Chemical Formula	RDBeq. value	*Δ* (ppm)	HRMS/MS fragment *m/z* (relative intensity)	Suggested Compound^1^
7.6	306.2429	C_19_H_32_O_2_N	4.5	0.928	288.23 (100), 227.18 (53), 253.2 (39), 271.21 (37), 245.19 (21), 241.2 (17), 259.21 (14), 262. 22 (12), 201.16 (11)	Samandarine
8.4	304.2273	C_19_H_30_O_2_N	5.5	1.033	286.22 (100), 304.23 (18), 243.17 (8), 269.19 (7), 217.16 (3)	Samandarone
8.4	348.2532	C_21_H_34_O_3_N	5.5	0.372	330.24 (100), 227.18 (47), 253.20 (35), 271.21 (32), 288.23 (25), 241.2 (23), 319.23 (18), 259.21 (18), 245.19 (14), 270.22 (13), 201.16 (11), 304.23 (10)	*O*-acetylsamandarine
8.5	292.2637	C_19_H_34_ON	3.5	0.609	292.26 (100), 147.12 (18), 161.13 (16), 291.25 (15), 135.12 (13), 105.07 (13), 145.1 (12), 121.1 (12)	Samanine
8.8	346.2378	C_21_H_32_O_3_N	6.5	0.450	328.23 (100), 299.2 (50), 239.18 (32), 259.17 (29), 317.21 (22), 257.19 (20), 285.19 (18), 311.2 (17),	Samandaridine
8.95	376.2846	C_23_H_38_NO_3_	5.5	0.450	316.23 (100), 358.24 (94), 376.28 (42), 332.26 (15, 287.29 (15), 196.14 (13)	Unknown samandarine
9.2	392.2797	C_23_H_38_O_4_N	5.5	0.294	241.2 (100), 259.21 (65), 374.27 (46), 227.18 (29), 288.23 (24), 253.2 (18), 271.21 (15), 199.15 (12), 270.22 (10), 215.18 (9)	*O*-3-hydroxybutanoylsamandarine
9.4	378.3003	C_23_H_40_NO_3_	4.5	0.530	378.3 (100), 274.25 (70), 377.29 (60), 334.27 (30), 360.25 (10)	Unknown samandarine
9.84	478.3163	C_27_H_44_NO_6_	6.5	0.428	288.23 (100), 460.31 (13), 374.27 (7), 270.22 (7), 241.2 (6), 434.29 (1)	Unknown samandarine
10.33	434.2901	C_25_H_40_NO_5_	6.5	0.00	288.23 (100), 374.27 (78), 416.28 (62), 241.2 (56), 259.21 (52), 270.22 (24), 227.18 (22)	Unknown samandarine
10.4	290.2474	C_19_H_32_ON	4.5	0.479	290.25 (100), 159.12 (11), 133.1 (10), 287.29 (10), 147.12 (9), 145.1 (9), 229.2 (7)	Samanone
10.76	374.2690	C_23_H_36_NO_3_	6.5	1.816	241.2 (100), 259.21 (97), 356.26 (52), 288.23 (43), 227.18 (34), 270.22 (25), 253.20 (22)	Unknown samandarine

^1^ Tentative identification

**Fig 5:**
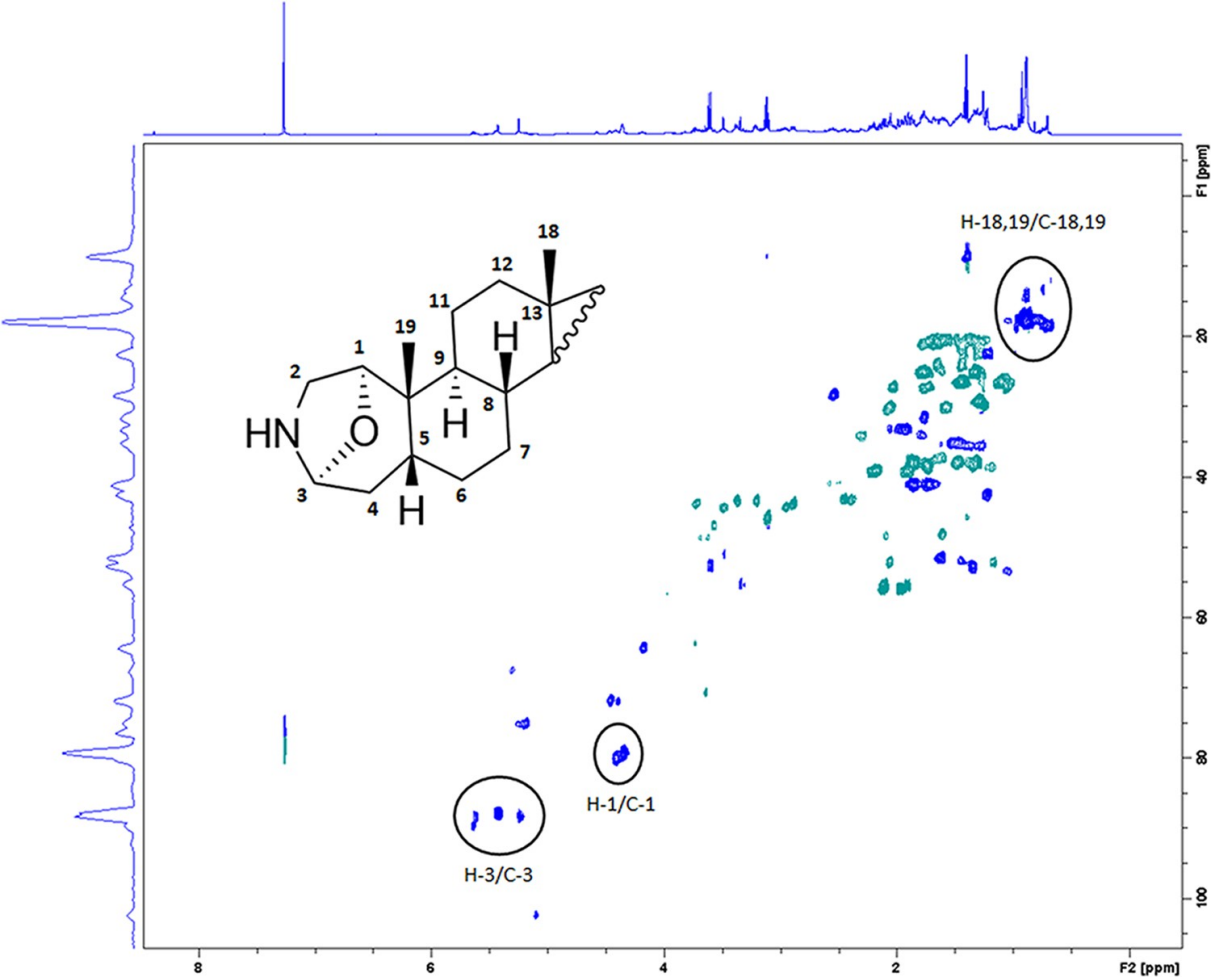
HSQC-DEPT (600 MHz, CDCl_3_) spectrum obtained from a pooled secretion sample.

In parallel to LC-MS analysis, a preliminary NMR analysis of all salamander species samples was attempted. The low excretion yield in *Lyciasalamandra* samples unfortunately did not allow meaningful structural information in the individual samples due to sensitivity limitations of NMR. However, it was possible to identify the basic samandarine skeleton and especially the oxazolidine spin-system combining 1 and 2D NMR spectra especially in the *S*. *salamandra* sample, given the higher secretion yield. Steroidal alkaloids of the salamander-type present in the secretions of *Lyciasalamandra* spp. were identified in the pooled sample of all specimens. In the ^1^H NMR spectrum, the presence of the steroidal moiety is evident by the singlet peaks present in the region between 0.86 and 0.96 ppm, corresponding to the characteristic protons of the methyl moieties on position 18 and 19. In the HSQC-DEPT spectrum (Fig 5), the corresponding methyl carbons resonate at approximately 17 ppm, consistent with the terminal methyl groups of a tetracyclic steroid terpene [47]. The presence of analogues bearing an oxazolidine system is especially evident in the HSQC-DEPT NMR spectra, by the characteristic cross-peaks corresponding to the protons and carbons of positions 1 and 3. Specifically, H-3 protons resonate as broad singlets in the region 5.00–5.80 ppm, while the corresponding carbons resonate in the range of 86.0 till 90.0 ppm. The deshielding of both H-3 and C-3 is indicative, being interpolated between a nitrogen and an oxygen atom, thus forming the characteristic oxazolidine system of samandarines. Concerning the rest of the oxazolidine ring signals in the HSQC-DEPT spectrum, the oxygenated methine C-1 and the corresponding H-1 resonating respectively at 80.0 and 4.40 ppm, as well as the cross peaks corresponding to the methylene groups of the position 2, in higher fields, are evident.

#### Other alkaloids

Many alkaloids other than samandarines, were also tentatively identified in the skin secretion samples (S1 Table in S1 File) using the same LC-HRMS/MS based dereplication and annotation workflow. Most of these alkaloids have previously been detected in skin extracts of various anuran genera [65] and trace their sources from various arthropods of their diet [58, 60, 66]. In order to elaborate on the possibility of dietary alkaloid sequestration in *Lyciasalamandra*, a bibliographic review was carried out to determine the ant and mite families present in the habitats occupied by the two *Lyciasalamandra* species, given that they usually represent the sources for most of these compounds. The dietary resources of Karpathos and Kasos share great similarity, while they differ from Kastellorizo [13]. An extensive catalog of ant species of the islands of Karpathos, Kasos and Kastellorizo exists, from many older or more recent censuses by many authors (e.g. Menozzi, Collingwood, Lopez, Forel, Finzi, Emery) (S2 Table in S1 File). A number of mite species from Karpathos and Kasos islands are also known, mainly from the Orders of Oribatida and Prostigmata (S3 Table in S1 File).

#### Population variance based on untargeted metabolomics

In order to delve further into the skin secretion metabolome of the Aegean salamander, in the present work, a metabolomics approach by means of untargeted UPLC-HRMS analysis was employed, so as to understand aspects of the regional and species differentiation of real salamander populations, according to their skin metabolome variability.

**Fig 6:**
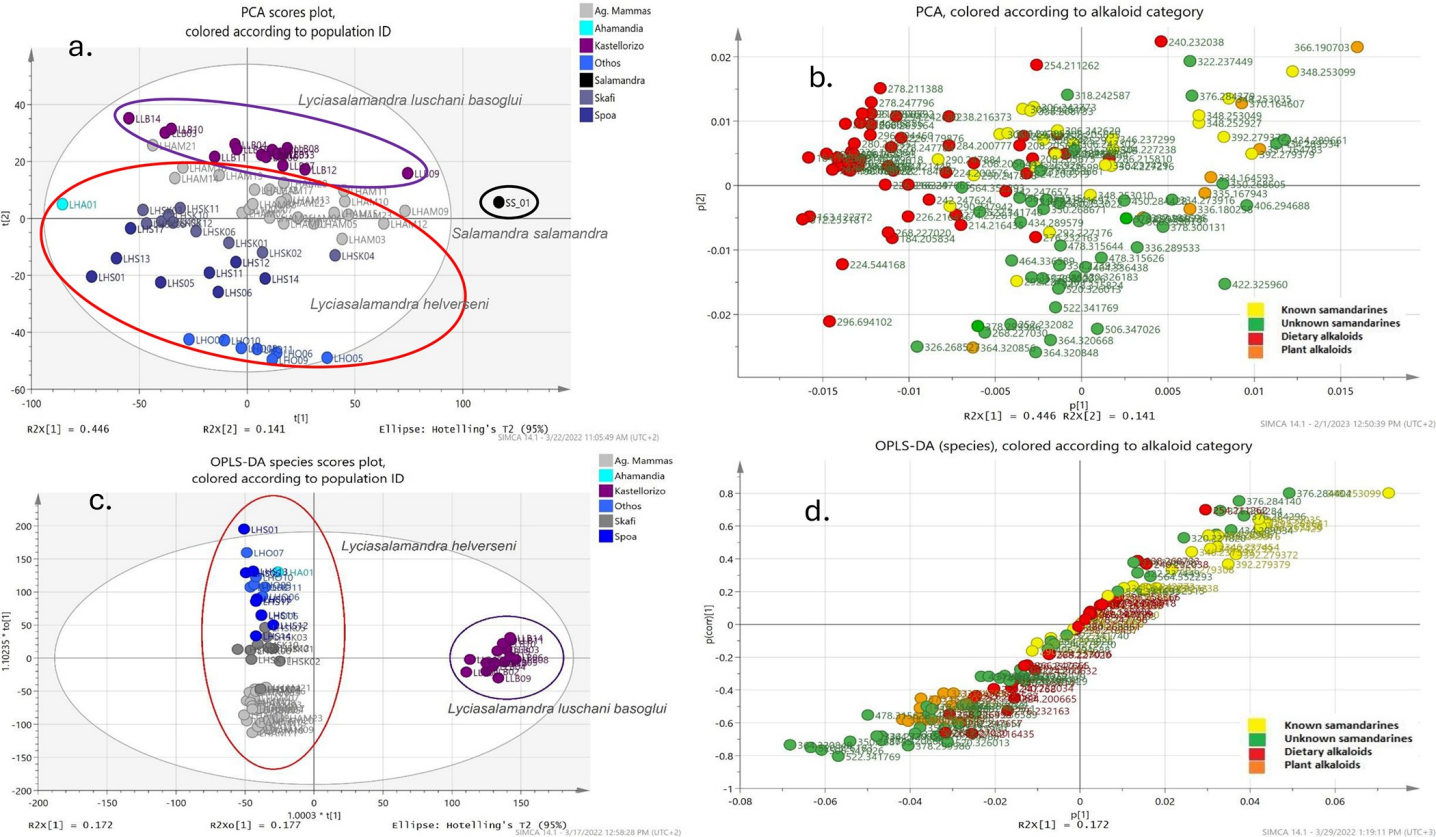
(a) PCA scores plot performed on the UPLC-HRMS data from the skin secretion samples of all 6 localities and *Salamandra salamandra* ssp. *salamandra* (SS); the first two principal components (PCs) explain 58.7% of the total variance (R^2^X = 76.7%, Q^2^ = 67.5%). (b) PCA loadings plot. Loadings shown represent putatively annotated compounds. (c) Scores plot of the OPLS-DA model obtained, classes according to species (R^2^X = 55.2%, R^2^Y = 98.9%, Q^2^ = 97.6%). (d) S-plot of the OPLS-DA model. Loadings shown represent putatively annotated compounds.

Principal Component Analysis (PCA) and Partial Least Squares–Discriminant Analysis (PLS-DA) was applied to all samples (LH and LLB) including the SS sample as reference, in order to investigate possible differentiation and classification trends and patterns (Fig 6A). After outlier identification, 65 samples were subsequently used for further processing. PC1 and PC2 accounted for 58.7% of the total variance in the dataset (Fig 6A). The permutation tests performed in the corresponding PLS-DA models (S2-S4 Figs in S1 File) supported the validity of the model (the intersection of R2 and Q2 regression lines with the vertical axis resulted in near zero and negative values, respectively). To further explore the metabolome constituents that play a key role in the differentiation of samples from the two *Lyciasalamandra* species, OPLS-DA was also performed, utilizing a dataset of 2520 variables with VIP scores larger than 1.0, produced from the PLS-DA analysis.

By considering the original data set with all variables (n = 7516), PCA revealed some degree of separation; The first PC does not clearly separate the samples, neither in terms of populations, nor in terms of other groupings (islands, species). So, most of the total variance (~ 45%), does not seem to be distributed among the various biogeographical units at first. However, ANOVA analysis of PC1 scores, in terms of populations, showed a statistically significant (P = 0.00049) differentiation (S5 Fig in S1 File). This indicates grouping in the first PC, albeit elementary, due to differences in means rather than dispersion, which is not the case for other biogeographic units (islands, species, P>0.05).

A regression analysis was performed: a) between scores of the first four PCs and peak height values of the various known and unknown samandarines plus SVL, as well as: b) between samandarine peak heights and SVL (S6 Fig in S1 File). Regression (a) revealed that a percentage of variation in the values of PC1 scores is explained by the variables samanone (10%), putative samandarines with *m/z* 378.3003 (C_23_H_40_NO_3_, RDBeq. 4.5), 434.2902 (C_25_H_40_NO_5_, RDB 6.5), 478.3160 (C_27_H_44_NO_6_, RDBeq. 6.5) (6–8%), *O*-acetylsamandarine (20%), *O*-3-hydroxybutanoylsamandarine (30.5%) and to a large extent by size (SVL, 55.8%). So PC1 is related to the fluctuation of some samandarines values, which in turn are related to the escalation of size of the individuals (from 7.5–38%, regression analysis (b)), probably mirroring developmental differences in samandarine production during the animal’s life. In contrast, PC1 loadings plot (Fig 6B) reveals that all alkaloids, other than samandarines have negative values. Also, along with PC1 values depending heavily on SVL gradation, most of the smallest specimens—regardless of origin—occupy the negative half of PC1 scores plot. According to the loadings plot, samandarines have a large contribution in the first PC, with *O*-acetylsamandarine, *O*-3-hydroxybutanoylsamandarine, and unknown samandarines having major contributions.

PC2 population separation is more obvious, as well as island and species, revealed by ANOVA (S5 Fig in S1 File). Regression analysis in PC2 shows that about 29% of the variance of scores is explained by the independent variable *O*-acetylsamandarine. In the loadings plot, it is obvious that *O*-acetylsamandarine, *O*-3-hydroxybutanoylsamandarine, and unknowns such as *m/z* 376.2846 (C_23_H_38_O_3_N, RDBeq. 5.5) characterize LLB, while samanine and a plethora of unknown samandarines characterize LH. Also, a cluster of compounds (S4 Table in S1 File) that characterize larger sized individuals, are diagnostic of Kasos Island populations. Most non samandarine type alkaloids display a disseminated distribution, which is individual specimen driven, rather than regional.

In PC3, there is a statistically significant differentiation of scores means of all biogeographical units (ANOVA), though the groupings do not relate to phylogeny (Othos—Kastellorizo). In PC4 there is no clear clustering, although populations of Othos and Kastellorizo display similar grouping, as in PC3. Nevertheless, the analysis of variance showed a statistically significant difference in the averages of population scores.

**Fig 7:**
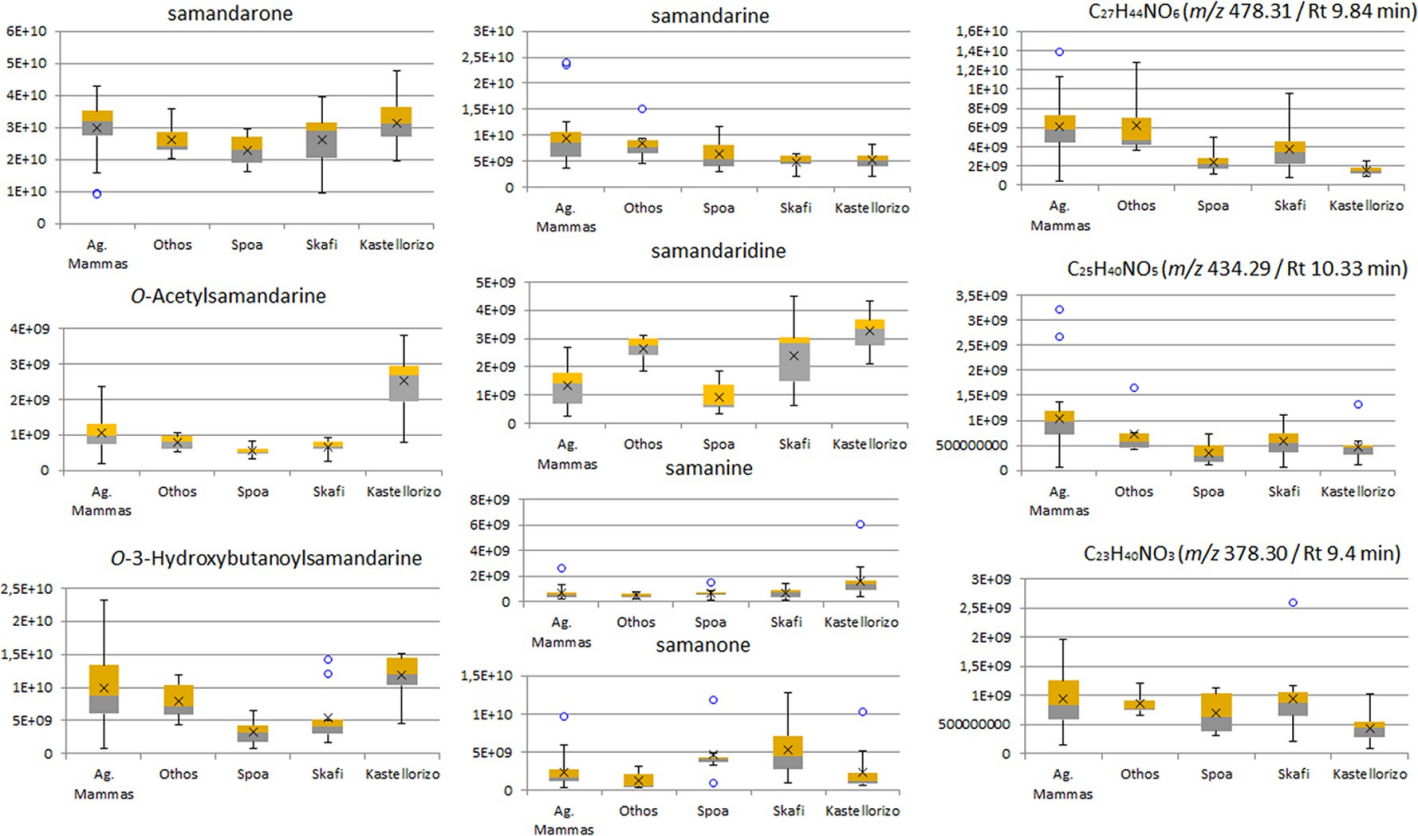
Boxplots of samandarine content (expressed as calculated peak intensity for the tentatively annotated features) across populations. Center line: median, X: mean, Whiskers: max–min.

An ANOVA analysis was also performed, so as to assess the differences between biogeographic entities, regarding their content in various samandarines. The differences in mean values of all samandarines across populations (Fig 7) proved significant, with the exception of samandarone. Across islands, exceptions correspond to samandarine, samandarone and samanone while across species non-significant proved samandarone and samanone (P>0.05).

According to the OPLS-DA analysis (Fig 6C), a clear clustering between the two classes corresponding to species was observed. One predictive and three orthogonal components were taken into account for the model (*R*^2^*X* = 55.2%, *R*^2^*Y* = 98.9%, *Q*^2^ = 97.6%). These results are similar to the PLS-DA findings. According to the species OPLS-DA model, samandarines proved statistically significant regarding interspecific separation. The corresponding S-plot (Fig 6D) reveals that known samandarines (*O*-acetylsamandarine, *O*-3-hydroxybutanoysamandarine, samanine, samandaridine and more) characterize LLB, while many unknown samandarines and other alkaloids characterize LH. According to the PLS-DA (and OPLS-DA) scores plots, the samples originating from different islands are clearly differentiated, whereas in the case of subregions, Karpathos localities cluster together, and Kasos localities are separated (S3, S4 Figs in S1 File).

In particular, Kasos Island populations (Skafi and Agios Mammas) do not appear to cluster together in both PCA and OPLS-DA models, and differentiating parameters can be observed, such as the difference in samandarine alkaloids representation (Fig 6). Interestingly, certain diagnostic compounds can only be observed for the Ag. Mammas region, while other compounds appear to be diagnostic of the entire island of Kasos (S7 Fig in S1 File). A diagnostic compound of Kasos island with a very high VIP score (VIP: 3.5, [M+H]^+^ C_21_H_24_NO_5_, *m/z* 370.1647; S3 Table in S1 File), was tentatively identified as an isomer of plant aporphine, protopine and isoquinoline alkaloids (e.g. 7-hydroxydehydroglaucine, cryptopine, fumaricine), mostly from various Papaveraceae species like *Glaucium* spp., which are also part of the S. Aegean flora (e.g. *Glaucium flavum*, *Glaucium corniculatum*, *Fumaria capreolata*). However, HRMS/MS spectra of the aforementioned [M+H]^+^ ion, produced from various Papaveraceae species analyzed (*Glaucium flavum*, *Fumaria* spp., *Papaver rhoeas*), did not identify with the corresponding *Lyciasalamandra* [M+H]^+^ ion spectrum. Best match produced by Sirius 5.8.0 [64] was the aporphine alkaloid Ocopodine (CSI:FingerID search score: -67,859).

In the same context, seven additional features, diagnostic of Kasos Island, were also identified belonging to the pyrrolizidine alkaloids chemical class (S3 Table in S1 File). All compounds presented in S3 Table in S1 File (with the exception of the aforementioned *m/z* ([M+H]^+^ 370.1647) were compared against those detected in a *Senecio vulgaris* specimen analyzed, and were found to have identical detected [M+H]^+^ and RDBeq, though the majority of the alkaloid content of the plant was the equivalent less toxic N-oxides. Annotation of the pyrrolizidine alkaloids Seneciphylline, Senecionine and Senkirkine was aided by Sirius 5.8.0 [64] (CSI:FingerID search score: -12,615, -9,753, -9,956 respectively). Additionally, in five LH specimens originating only from Kasos island the alkaloid coccinelline was also putatively identified [C_13_H_24_ON ([M+H]^+^ *m/z* 210.185)]. The comparison of full scan and HRMS/MS with the analyzed sample of the *Coccinela septempunctata* (seven-spot ladybug) indirectly verified its annotation (S8 Fig in S1 File).

## Discussion

Samandarine alkaloids are the most abundant alkaloids detected in the secretion metabolome of the samples obtained from Lycian salamanders. It is noteworthy that although the *Salamandra* alkaloids were discovered in 1866, isolated in the early 1930s [33] followed by a boom concerning their identification during the 1960s, very little literature occurs relative to the now established structure elucidation techniques such as NMR [47] and LC-MS. The oxazolidine moiety of the *Salamandra* alkaloids appears to be unique and specific not only to *Salamandra*, but also to other Salamandridae genera [8], including *Lyciasalamandra* species.

The application of LC-HRMS/MS metabolomics on the animal’s secretion metabolome provided new insight its alkaloid composition and permitted the detection of variations between different regional subdivisions. The observed differentiation among various taxa has multiple causes and contributing factors, being derived from a complex compound cocktail that affects many different aspects of the organism’s homeostasis [39, 45]. In turn, it is influenced by a wide and diverse array of environmental and genetic factors and processes, thus obscuring of course any phylogenetic information [8, 9, 12]. The compound content of the exocrine metabolome of Lycian salamanders is differentially affected by genetic drift (small, isolated populations [11]), natural selection and regulation (permanent or incidental differences in secretion content, based on response to environmental variables) being protein mediated. Environmental variables can include, for example, predator presence, inflammation, diet, seasonal changes, etc. In fire salamanders, it is well known that samandarine ratios alter with time, thus regulation of the secretory product seems plausible [57]. Mucosome also affects the contents of salamander secretions (interactions between the secretory product and the skin microbiota could alter regionally its contents [45]).

Taxon subdivision in MVA can be therefore attributed to a multitude of factors affecting the chemical content of the various populations’ skin secretions, given that in this case, most of the skin secretion metabolome is used in the analysis. Moreover, the populations considered are characterized by known differences in genetic profiles and enhanced isolation. Regional differences in various samandarines content are even harder to explain -since the particular effects of each are unknown- so as to correlate their relative abundance to specific factors, especially after the detection of many more different variants (samandarines have been also implicated as a means of chemical signaling, connected to site fidelity or other signals) [67]. In any case, metabolomics can provide a comprehensive toolbox towards the investigation of parameters affecting populations with divergent metabolomes, by controlling for different candidate underlying factors.

Especially in the case of the two closely residing populations of Kasos island (Skafi and Agios Mammas), which belong to the same species and are separated by 2.62 km in a straight line, the shortest distance between phylloclades found in the genus [10], several differences were disclosed based on the MVA performed on the dataset. Note that the whole species has three phylloclades, two of which are found exclusively in this area. Moreover, Skafi population specimens bear three mtDNA haplotypes, private to this population, indicating long term isolation. Sequencing of Cytb and 16S mtRNA gene fragments, revealed a considerable amount of divergence (1.8% and 0.4% for Cyt b and 16S, respectively) [4] which corresponds to a separation time of more than 0.5 mya [4], though the calibration point used for the molecular clock was far later than the one accepted today [11], thus moving the separation time a great deal earlier. This is mostly due to an intermediate area of non-limestone bedrock, which impedes the two populations from coming to contact (S1 Fig in S1 File). The two populations, even though extremely adjacent, inhabit a slightly different habitat type, so we can assume variation of at least some environmental features and / or microclimate, which in conjunction with long—term isolation, could partly explain the observed diversification of their secretion components (e.g., samandarines). Different adaptations to predation or pathogens cannot be assumed as a plausible cause of regional diversification, given the sheer vicinity and relative homogeny of environmental factors affecting the two populations of Kasos.

Similar assumptions can be made for the two populations collected in Karpathos. The Othos population occupies an area in the center of the island of Karpathos, with an altitude of around 500m. It seems recently and partly isolated (under HW disequilibrium [4]) in a favorable microclimate, dictated by a limestone substrate (S1 Fig in S1 File), and increased relative humidity. It belongs to the same phylloclade as the Spoa population, though possesses a quite divergent haplotype [10]. This population however, appears to be the most diversified in the first PCA components, while the PLS-DA and OPLS-DA species models cluster the Othos population with the rest of Karpathos populations (Fig 6C and S5 Fig in S1 File). Spoa population on the other hand, resides on a slope under Lastos mountain, with tall vegetation present (*Pinus brutia*), calcareous substrate and a water spring, flooding stone terraces downstream. It does not seem as isolated and is surrounded by suitable habitat as well as other known LH populations.

The case of environmental parameters causing diversification of the exocrine secretions among populations, has been previously investigated in other amphibians, taking into account alkaloid profiles [60]. It has been shown that various amphibians are able to sequester dietary alkaloids, while the availability of alkaloid-containing arthropods emerged as a differentiating factor for alkaloid profiles of neighboring populations or different species [68]. Nevertheless, this does not rule out genetic variation as a discriminating factor, as alkaloid sequestration is protein mediated [69]. In poison dart frogs, the percentage of dietary ants is positively correlated with the amount of alkaloids sequestered in the skin [70], so it is straightforward to assume that differences in the overall composition of an individual’s diet concerning alkaloid bearing arthropods, has direct effects on the skin alkaloid composition. In the case of *Lyciasalamandra* of course, samandarine alkaloids, being the dominant compounds in the secretion, play the most significant role in diversifying the studied populations (Fig 7). Being internally synthesized, they are gene regulated and their expression is probably affected by certain environmental factors, that remain to be determined.

In some cases though, regional diversification of *Lyciasalamandra* populations can be partly attributed to other compounds, that appear to be diagnostic of particular populations (e.g., diagnostic alkaloids of Kasos, S7 Fig in S1 File). HRMS-MS/MS analysis resulted in the putative identification of a large number of non-internally biosynthesized alkaloids in the secretions of Lycian salamanders (S1 Table in S1 File), although alkaloid sequestration in *Lyciasalamandra* cannot be verified beyond doubt by the evidence presented herein alone. According to our findings, we present indications of sequestration of alkaloids in Lycian salamander species, such as PTXs, coccinellines decahydroquinolines, substituted piperidines, pyridine and indole alkaloids (S1 Table in S1 File). In most cases, these alkaloids are found in trace amounts and in some instances only in a small number of individuals from a given population. For example, ladybird alkaloids, both precoccinelline and coccinelline (S8 Fig in S1 File), as well as tricyclic 207GH/K were found in just five individuals in the Ag. Mammas population of Kasos Island (*L*. *helverseni*). These alkaloids are normally found in poisonous anuran species of the genera Dendrobatidae, Bufonidae, Mantellidae, Myobatrachidae and Eleutherodactylidae of the Southern hemisphere. All these alkaloid groups have a proven or speculated origin from consumption of arthropod prey by the predator amphibians. Most of them originate from oribatid mites, coccinellid beetles, myrmicine and formicine ants [18, 58, 71, 72] while the indole and pyridine alkaloids (nicotine, noranabasamine, pyridylnicotine, calycanthine and chimonanthine) probably originate from plant material, with arthropod consumers as intermediate carriers [59, 73]. PTXs and Allopumiliotoxins are known to originate from oribatid mites and formicine ants, and have been found in the ant genera *Brachymyrmex*, *Paratrechina* and *Nylanderia* [74, 75]. Representatives of all arthropod taxa mentioned above are established fauna of the islands (S2, S3 Tables in S1 File). This fact corroborates that, in the present case, the alkaloids identified besides samandarines, might be sequestered in a similar manner to many anuran groups containing noxious skin chemicals.

*Lyciasalamandra* species have very diverse feeding habits, consuming a wide range of invertebrates, namely gastropods, annelids, isopods, myriapods, arachnids (mites, pseudoscorpions, spiders) and many orders of insects [76–78]. *Lyciasalamandra* species’ diet, depends heavily on availability during different seasons and / or localities, as can be inferred from differences found between various references [76, 78]. No statistically significant differences were found between male and female % prey item consumption [76, 77] nor between seven species of *Lyciasalamandra* in Turkey [78]. Concerning insects, the most consumed prey items in the case of most *Lyciasalamandra* species (including *L*. *luschani* and *L*. *helverseni*), belong to the orders Coleoptera, Hymenoptera, Diptera, Dermaptera, Collembola, Heteroptera and Lepidoptera. Certain Lepidoptera species larvae along with several Coleoptera, sequester pyrrolizidine alkaloids, sourced from various plant species of Boraginaceae, Fabaceae (tribe Crotalarieae) and Asteraceae (tribe Senecioneae) [79], which they also utilize as pheromones [80] (e.g. senecionine, seneciphylline, senkirkine). These appear to be, among others, the Kasos diagnostic plant derived alkaloids (S4 Table in S1 File). Species of all aforementioned families are part of the flora of the southern Aegean Greek islands, while there are also Lepidoptera species whose larval food plants are Boraginaceae (e.g., *Utethesia pulchella*) or Senecioneae species (e.g., *Epiblema hepaticana*, *Zamacra flabelaria*, *Lacanobia oleracea*). It is known that insects that sequester pyrrolizidine alkaloids, which are probably the intermediary in this case between the plant and the amphibian, do so by storing, or converting back to the aforementioned less toxic N-oxides [81]. In the salamander’s exocrine poison glands though, converting back to N-oxides is probably not the case. There was complete absence of any N-oxides in the salamander samples, thus compartmentalization is probably the way to avoid autotoxicity, as with other anuran species and alkaloids, whereas certain adaptations cannot be ruled out [82]. This, to our knowledge, is the first record of the particular pyrrolizidine alkaloid class being sequestered by any amphibian, however further research is needed to verify this hypothesis.

An interesting observation in PC1 loadings plot (Fig 6B), is the SVL-dependent distribution of putative dietary alkaloids: in small individuals concerning ant or mite derived alkaloids, or large individuals concerning pyrrolizidine plant derived alkaloids (S4 Table in S1 File), possibly acquired from lepidoptera larvae [13]. This could be an indication of prey item size correlation to salamander specimen size [69], taking into account that alkaloid containing arthropods are most often of small size (mites, ants).

In the case of alkaloid sequestration besides samandarine biosynthesis, *Lyciasalamandra* (and *Salamandra* for that matter) would appear to be another group of amphibians to contain internally synthesized alkaloids, as well as sequestered alkaloids from external sources, along with *Pheudophryne coriacea* [43].

This is of particular importance, due to the fact that there is no record of other urodeles sequestering alkaloids from their diet, which further imposes questions concerning the origin, common or multiple, of the compound sequestration mechanism, as well as the relationships between the prey items that constitute the source of the sequestered alkaloids, over a vast portion of the globe. On that matter, Clark *et al*. [83] state that similarities in alkaloid sequestration between Neotropical and Malagasy poison frogs, are a consequence of convergent evolution in the endemic ant radiation, followed by that of the molecular mechanism of the uptake system. A plausible scenario proposed for the development of the alkaloid sequestration system, is a three step evolutionary pathway, where at first, predator resistance to pray toxins evolves, followed by sequestration of the toxin by the predator, and lastly exploitation of the toxin for the defense of the predator itself [84].

Absence of some alkaloids from *S*. *salamandra* cannot be taken as sufficient proof of non-existence, because of the lack of significant specimen representation though size dependence of dietary alkaloid sequestration as presented above might mean that less of those alkaloids are to be found in bigger salamander species.

The possibility of alkaloid sequestration by *Lyciasalamandra* species, raises questions on the utilization and possible regulation of the alkaloid arsenal from different sources, produced and sequestered. The secretion of diet-derived alkaloids could represent merely added unpalatability, or rather achieve different functions, like what is suggested for *M*. *moreirae*, that might regulate bufotenine production in relation to the total quantity of sequestered dietary alkaloids [44], or *Pseudophryne* species, that possibly turn off biosynthesis of pseudophrynamine alkaloids when dietary alkaloids are abundant [43]. However, based on our findings, it seems that younger individuals of *Lyciasalamandra* species hold comparatively lower samandarine content and more dietary alkaloids. Predator–prey interactions, like possible predator toxin resistance, could also explain prey (salamander) toxin arsenal enrichment, either in the form of samandarine type alkaloid diversity, or sequestration from dietary sources, in a form of an evolutionary arms race. The functions served by each compound and the level of regulation in these systems are still to be determined.

## Conclusions

In conclusion, we report herein a preliminary investigation of the skin secretions of *Lyciasalamandra* species collected in Greece. UHPLC-HRMS metabolomics and MVA revealed differentiation of populations, attributed to the profile of secondary metabolites and alkaloids. This diversification is not consistent with the phylogenetic structure of studied populations. Important parameters for the differentiation included the presence of samandarines and their levels in the selected populations, while they were identified for the first time in LH and LLB. According to UHPLC-HRMS/MS profiling, a large number of unidentified compounds were also detected, belonging to the same family. Additionally, we provide preliminary evidence of dietary alkaloid sequestration for both *Lyciasalamandra* species and *S*. *salamandra*, the first to our knowledge among urodele Amphibians. Those evidence were generated by partial characterization or annotation of compounds (alkaloids) most of which are known to be sequestered by other amphibians, while a group of plant derived pyrrolizidine alkaloids, known to be sequestered by Lepidoptera larvae, are for the first time detected in the skin secretion metabolome of any amphibian.

Further research should focus on isolation and characterization of novel samandarines present in the skin secretion of *Lyciasalamandra* and *Salamandra* species, as well as combined sampling and analysis of salamander melabolomes, their stomach contents, arthropod fauna and local plant species of certain population localities, so as to better understand if a path exists, from plant compounds to arthropod pray, all the way to skin granular glands of salamanders. Further employment of metabolomics can help pinpoint which parameters affect certain parts of the skin secretion metabolome, towards a better understanding of its various functions.

## Supporting information

S1 File Supporting figures and tables. (DOCX)

S2 File LC-HRMS metabolomics datasheet. Peak height values are normalized by animal weight.
(XLS)

S3 File Snout to Vent Length (SVL) values for sampled animals. (XLSX)
