# Supplementary material for: A skin secretion metabolome analysis of the Greek Dodecanese Lycian salamanders: Preliminary evidence of dietary alkaloid sequestration in urodeles
Source: PLoS One. 2024 Aug 29;19(8):e0300278. doi: 10.1371/journal.pone.0300278 (PMC11361651; doi:10.1371/journal.pone.0300278)
Supplement: S1 File — (DOCX) [file pone.0300278.s001.docx]

**Supporting Information**

**A skin secretion metabolome analysis of the Greek Dodecanese Lycian salamanders: Preliminary evidence of dietary alkaloid sequestration in urodeles.**

Karolos Eleftherakos^1^, Roza Maria Polymeni^1^, Eleni V. Mikropoulou^2^, Konstantina Vougogiannopoulou^2^, Christos Georgiadis^1^, Eleftherios A. Petrakis^2^, Leandros A Skaltsounis^2^, Maria Halabalaki^2^*

^1^ Section of Zoology – Marine Biology, Department of Biology, National and Kapodistrian University of Athens, Panepistimiopolis Zografou, Athens, Greece

^2^ Division of Pharmacognosy and Natural Products Chemistry, Department of Pharmacy, National and Kapodistrian University of Athens, Panepistimiopolis Zografou, Athens, Greece

* Corresponding author:

Email: mariahal@pharm.uoa.gr


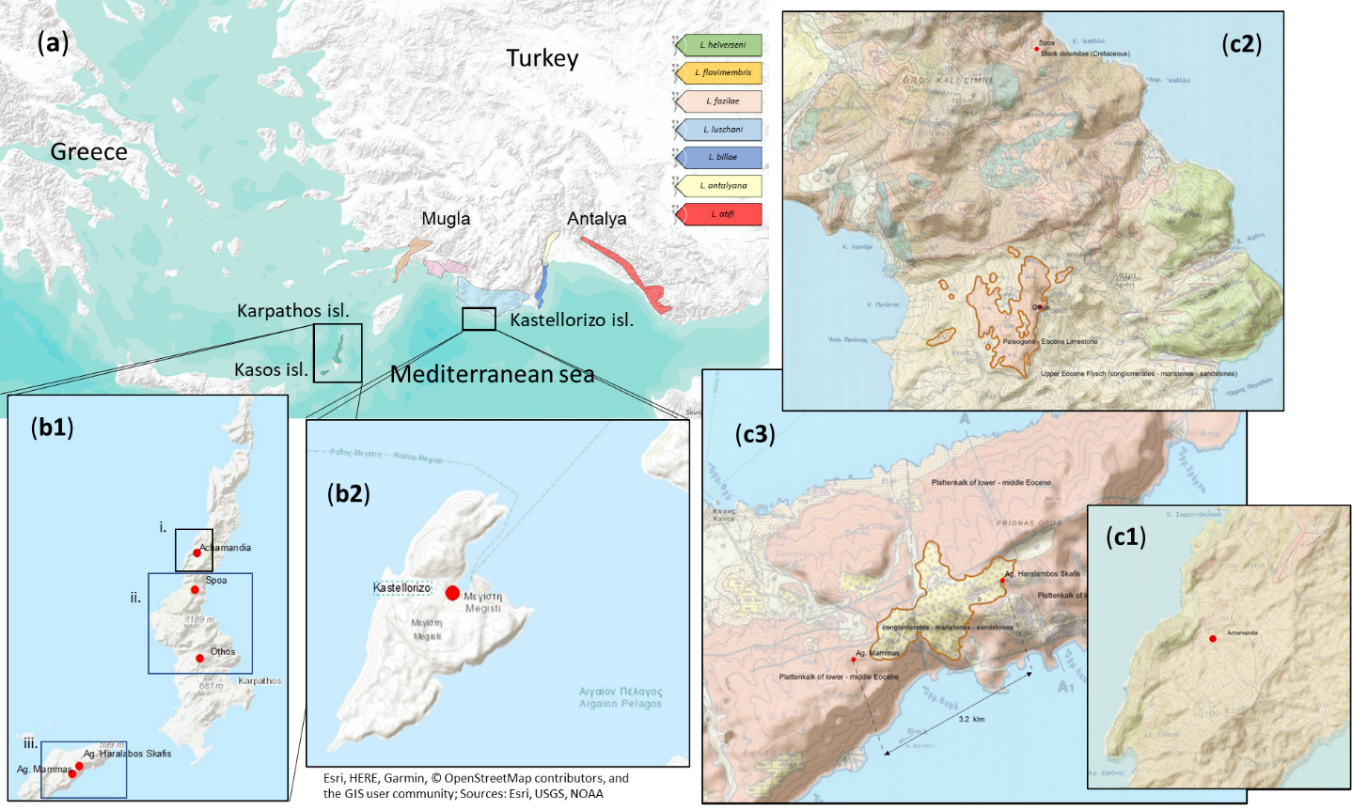


**S1 Fig.** (a) *Lyciasalamandra* species geographical distribution map (after Veith *et al*. 2020). (b1,2) Sampling localities (*S. salamandra* not included). (c1,2) Karpathos Island, (c3) Kasos Island.


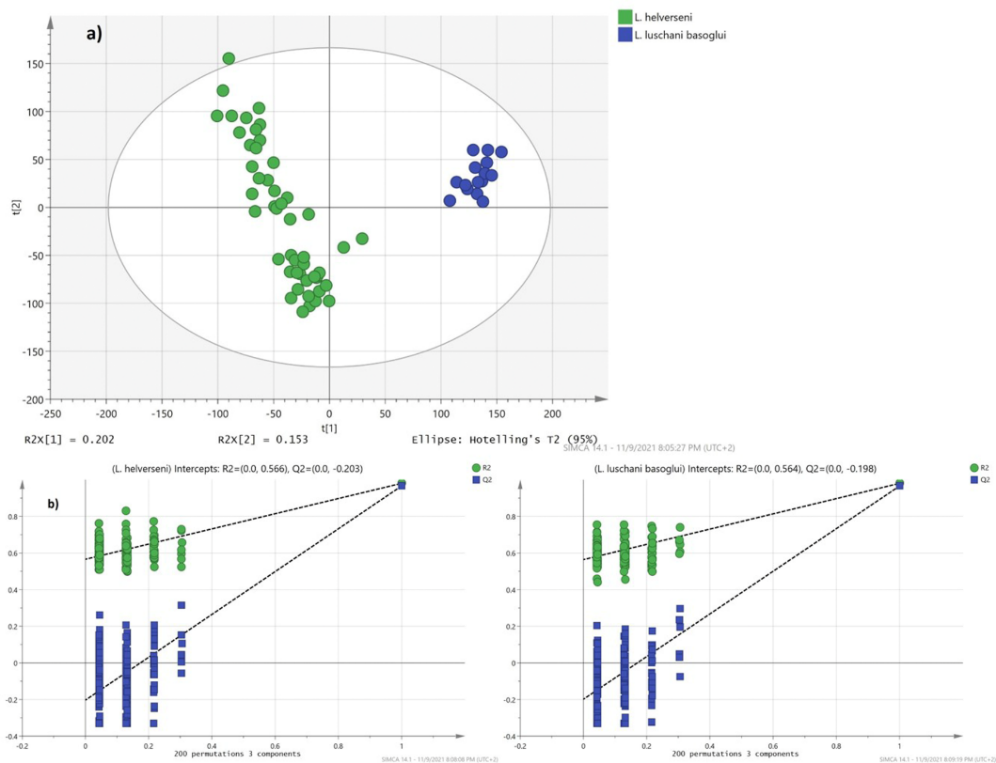


**S2 Fig.** Scores plot (a) and permutation test (200 random permutations) plots (b) of the PLS-DA model obtained from the UPLC-HRMS data of the skin secretions, considering the samples of *Lyciasalamandra helverseni* (LH) and *Lyciasalamandra luschani* ssp. *basoglui* (LLB) (*R*^2^*X* 65.1%, *R*^2^*Y* = 93.5%, *Q*^2^ = 89.9%).


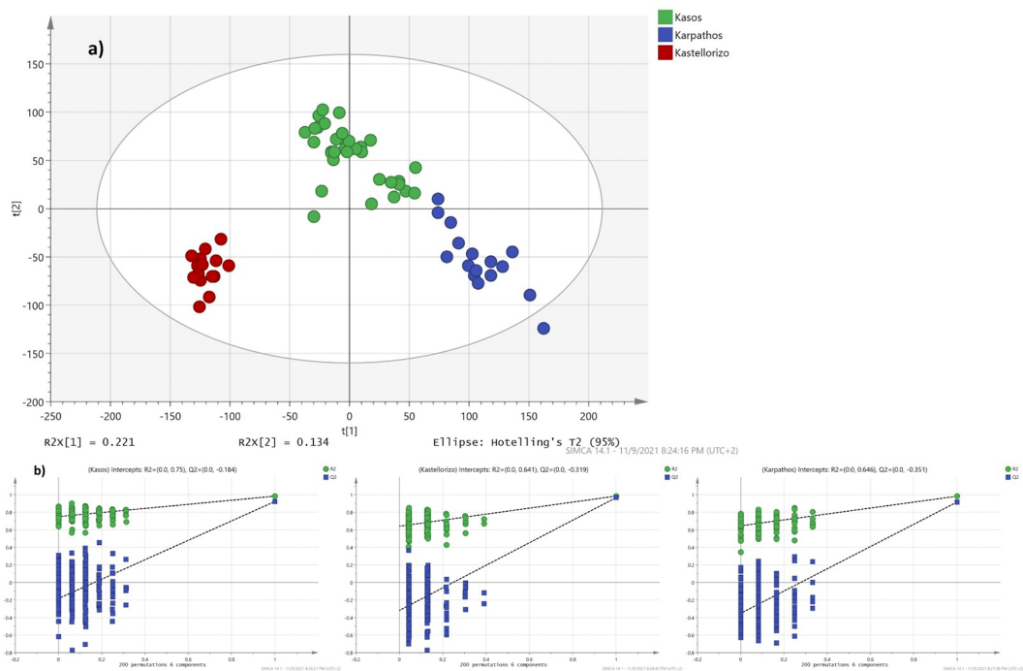


**S3 Fig.** Scores plot (a) and permutation test (200 random permutations) plots (b) of the PLS-DA model obtained from the UPLC-HRMS data of the skin secretions, considering the samples from the islands of Karpathos, Kasos, and Kastellorizo (*R*^2^*X* = 73.6%, *R*^2^*Y* = 93.3%, *Q*^2^ = 87.6%).


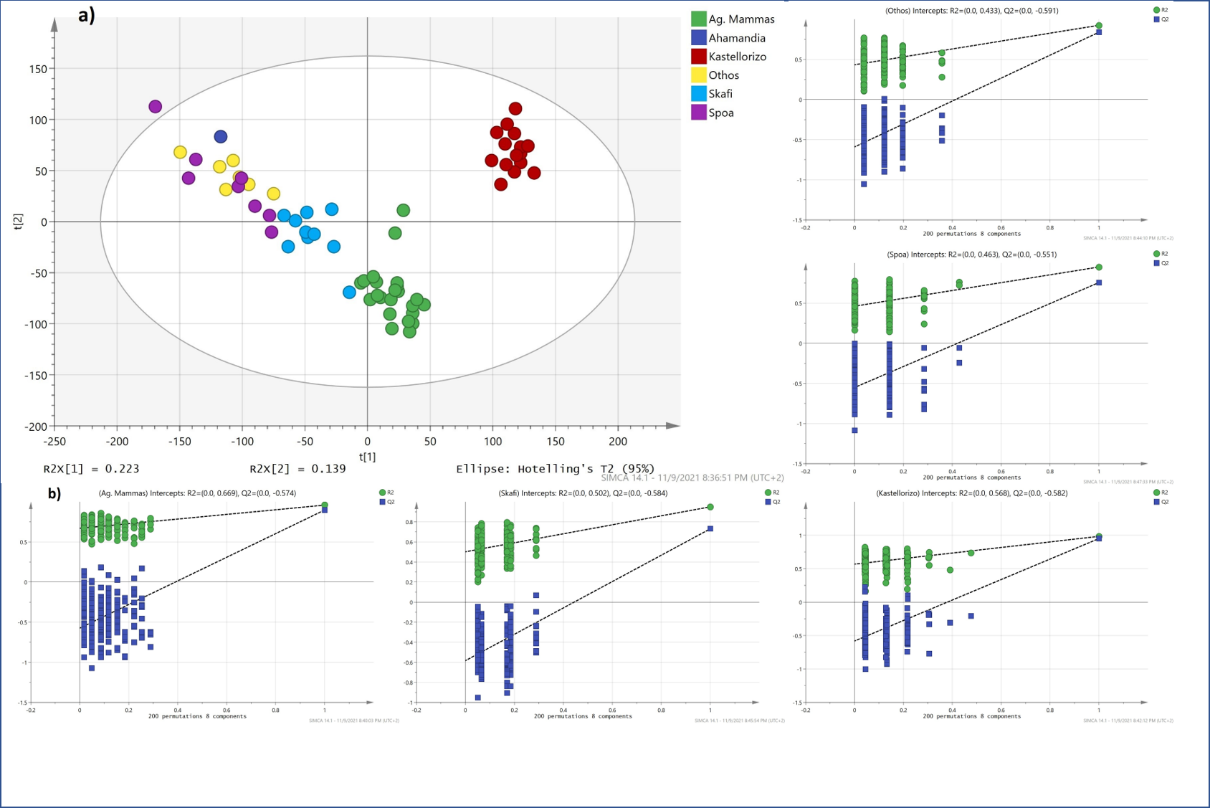


Kastellorizo

Kasos

Karpathos

**S4 Fig.** Scores plot (a) and permutation test (200 random permutations) plots (b) of the PLS-DA model obtained from the UPLC-HRMS data of the skin secretions, considering the samples from five different locations, namely Agios Mammas (Kasos), Kastellorizo (harbor; Kastellorizo), Othos (Karpathos), Agios Charalampos Skafis (Kasos), and Spoa (spring; Karpathos) (*R*^2^*X* = 75.0%, *R*^2^*Y* = 88.3%, *Q*^2^ = 79.6%).


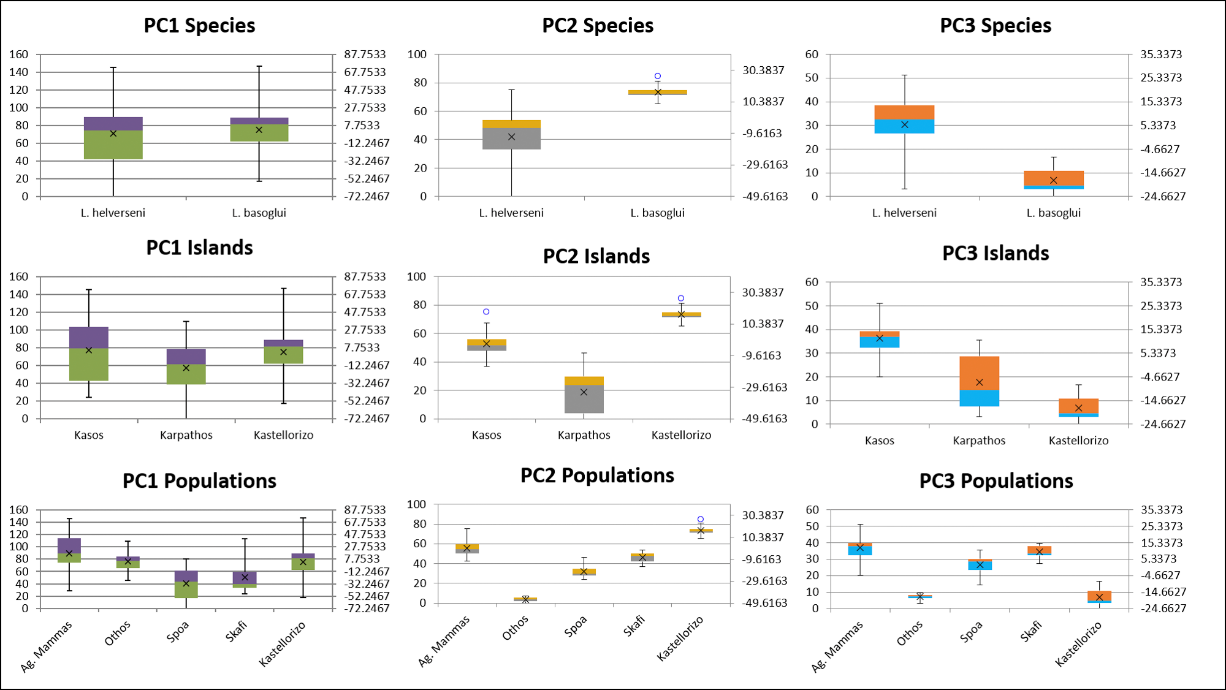


**S5 Fig.** PCA Scores boxplots for populations, islands, and species. Center line: median, X: mean, Whiskers: max – min.


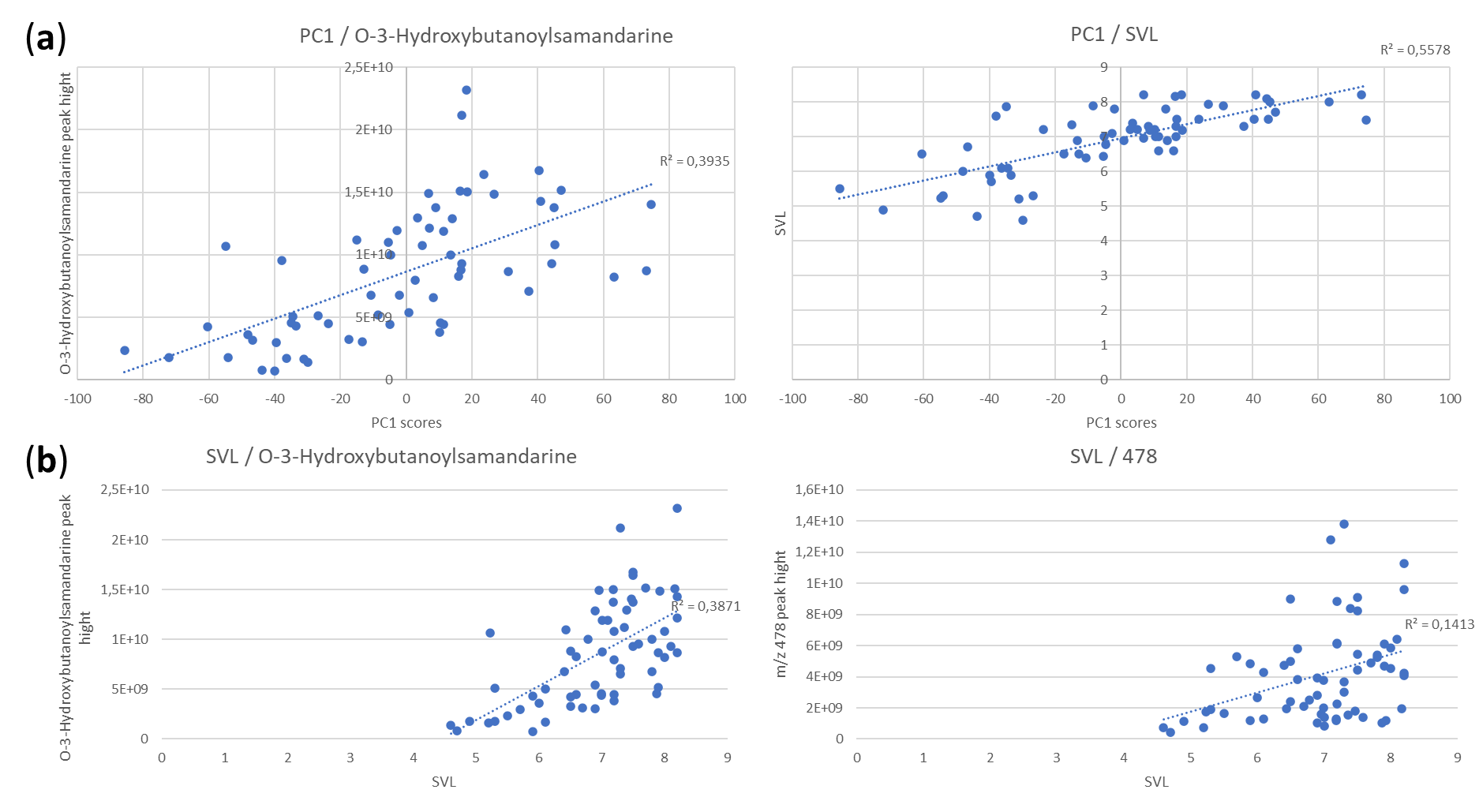


**S6 Fig.** Regression analysis a) between scores of the first four PCs and peak height values of the various known and unknown samandarines plus SVL, b) between samandarine peak heights and SVL


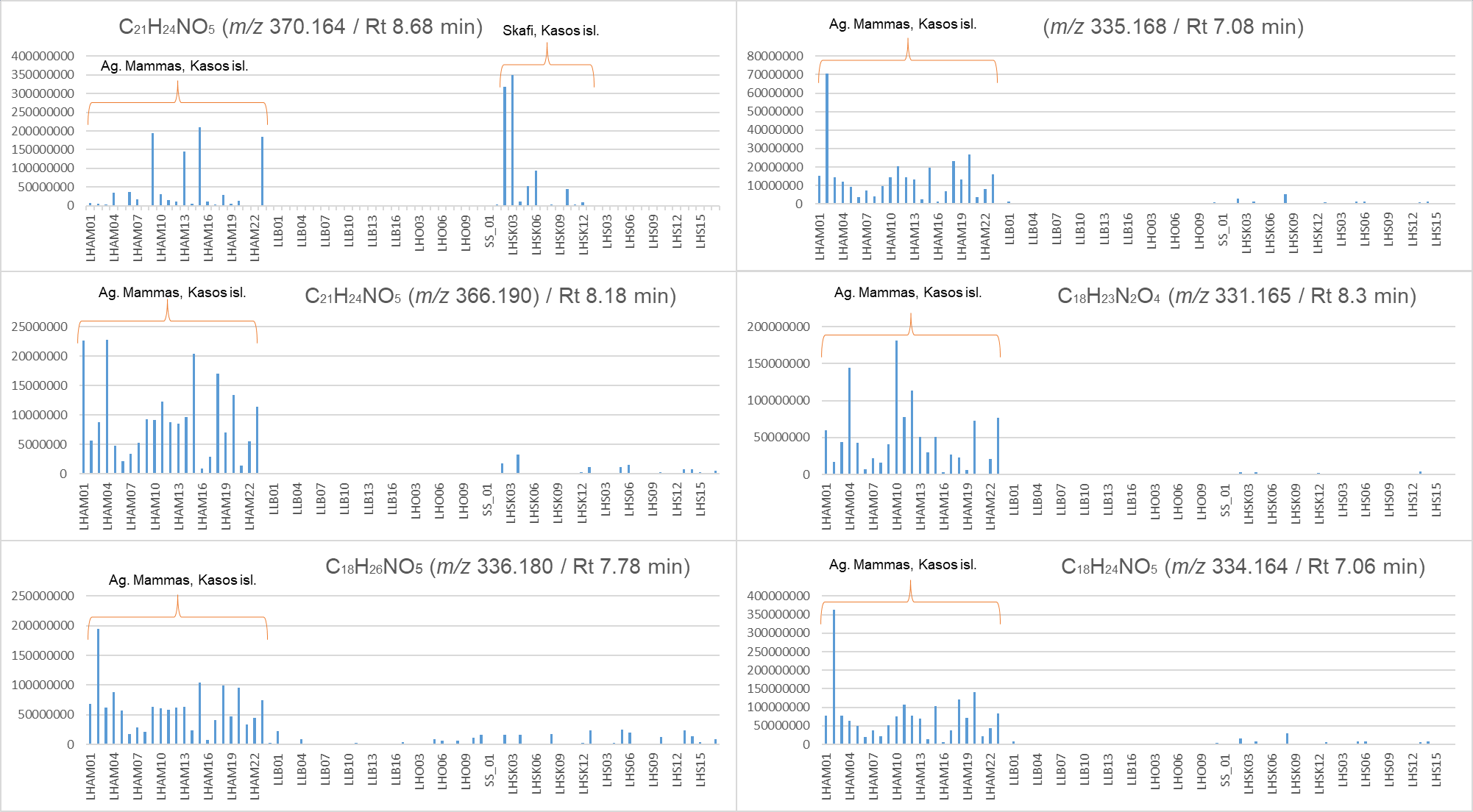


**S7 Fig.** Distribution of diagnostic compounds of Ag. Mammas population or Kasos Island.


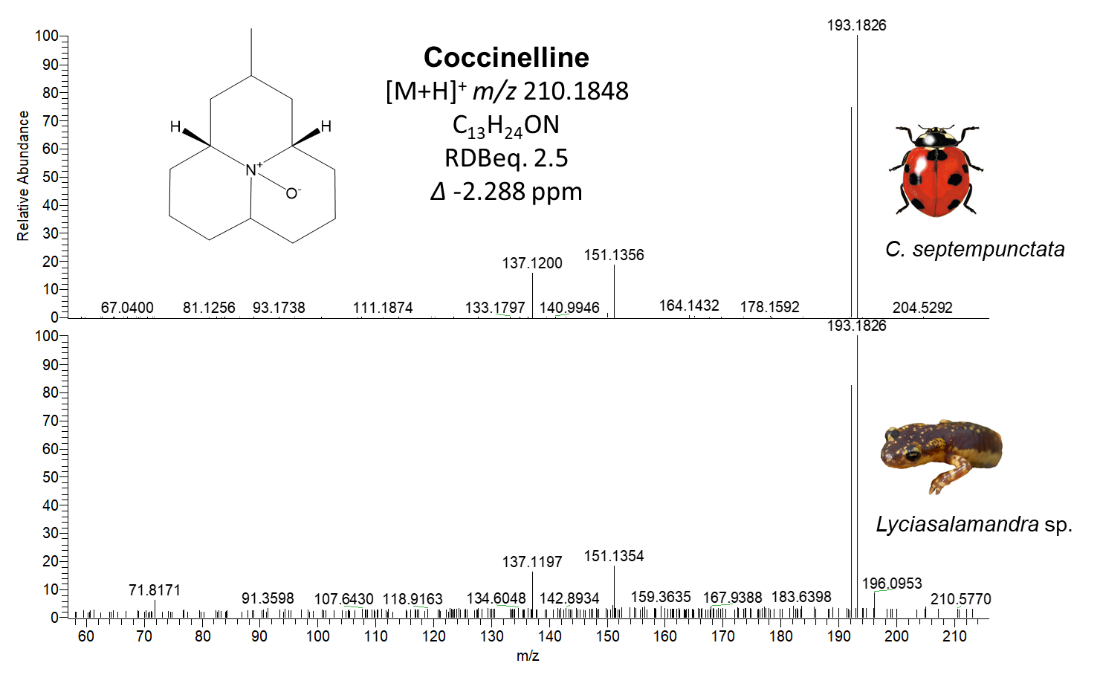


**S8 Fig.** Comparison of HRMS/MS spectra of the alkaloid coccinelline found in the skin excretions of LH samples and in a *C. septempunctata* sample.

**S1 Table.** Putatively annotated dietary alkaloids (only [M+H]^+^ ions).

| **AA** | **Retention time (min)** | **Experimental *m/z*, [M+H]^+^** | **Suggested molecular formula** | **RDBeq. values** | ***Δ* (ppm)** | **HRMS/MS fragment *m/z* (relative intensity)** | **Suggested Compounds^1^** | **Chemical group** |
| --- | --- | --- | --- | --- | --- | --- | --- | --- |
| 1 | 1.14 | 163.1227 | C_10_H_14_N_2_ | 4.5 | -0.055 |  | Nicotine | Alkaloids |
| 2 | 11 | 184.1695 | C_11_H_22_ON | 1.5 | 0.209 | 167.30 (100), 71.82 (92), 194.68 (82) | 3-5-disubstituted pyrrolizidine (3-(5-Methylhexahydro-1H-pyrrolizin-3-yl) propan-1-ol) | Alkaloids |
| 3 | 9.46 | 184.2061 | C_12_H_26_N | 0.5 | 0.124 | - | 2-5-disubstituted pyrrolidine 183B | Alkaloids |
| 4 | 7.22 | 194.1904 | C_13_H_24_N | 2.5 | 0.104 | 138.07 (100), 194.19 (48), 196.08 (30), 71.83 (15), 196.16 (9), 167.14 (8) | Precoccinelline, 5,8 disubstituted indolizidine 193E, Octahydroquinoline 193, 8-Deoxypumiliotoxin 193H, 9-Deoxy-hPTX 193h | Alkaloids |
| 5 | 7.90 | 198.1853 | C_12_H_24_ON | 1.5 | 0.229 | 198.19 (100), 196.30 (30), 107.09 (22), 156.97 (21), 196.32 (18), 95.09 (16), 196.35 (15), 179. 99 (15), 121.1 (15), 71.82 (13) | 5,8, Disubstituted indolizidine 197C, 5,6,8-Trisubstituted indolizidines 197G, H | Alkaloids |
| 6 | 9.79 | 198.2214 | C_13_H_28_N | 0.5 | 0.194 | - | 2-5-disubstituted pyrrolidine 197B, 2-6-disubstituted piperidine 197E, F | Alkaloids |
| 7 | 7.56 | 206.1899 | C_14_H_24_N | 3.5 | -0.616 | - | 5,8-Disubstituted Indolizidines 205A, Dehydroizidine 205F/G, Coccinelline-like tricyclics 205B | Alkaloids |
| 8 | 5.81-9.18 | 208.2056 | C_14_H_26_N | 2.5 | -0.336 | 208.21 (100), 70.07 (90), 95.09 (71), 121.10 (65), 109.1 (61), 84.08 (59), 107.09 (51) | Tricyclic 207GH/K | Alkaloids |
| 9 | 6.50 | 210.1851 | C_13_H_24_ON | 2.5 | 0.119 | 192.17 (100), 193.18 (23), 210.19 (13), 151.14 (5), 150.13 (5) | Coccinelline, | Alkaloids |
| 10 | 13.5 | 212.2008 | C_13_H_26_ON | 1.5 | 0.041 | - | (2S,5S)-5-methyl-2-propyldecahydroquinolin-6-ol (decahydroquinolin 211) | Alkaloids |
| 11 | 8.53 | 214.2167 | C_13_H_28_ON | 0.5 | 0.149 | - | 4-Hydroxy-2,6-Disubstituted Piperidine 213A, B | Alkaloids |
| 12 | 4.56-10 | 222.1849 | C_14_H_24_NO | 3.5 | -0.231 | - | 8-Dehydrodesmethylpumiliotoxin 221F | Alkaloids |
| 13 | 11.85 | 223.0638 | C_11_H_12_ON_2_Cl | 6.5 | 0.443 | 207.03 (100), 225.04 (21), 209.01 (13), 227.02 (1), 206.17 (1), 207.04 (1) | Phantasmidine | Alkaloids |
| 14 | 6.36-10 | 224.2005 | C_14_H_26_NO | 2.5 | -0.421 | - | PTX 223U, hPTX 223G | Alkaloids |
| 15 | 11.21 | 226.1802 | C_13_H_24_O_2_N | 2.5 | 0.114 | 208.09 (100), 180.11 (10), 184.97 (7) | Pumiliotoxin 225F, Allopumiliotoxin 225E | Alkaloids |
| 16 | 16 | 226.2164 | C_14_H_28_NO | 1.5 | -0.111 | - | 5,6,8-Trisubstituted indolizidine 225 K, L | Alkaloids |
| 17 | 11.40 | 226.2531 | C_15_H_32_N | 0.5 | 0.153 | 208.17 (100), 128.14 (23), 196.12 (21), 180.17 (18), 97.1 (16), 71.82 (14) | 2,6-Disubstituted piperidines 225B, 225I, 2,5-Disubstituted pyrrolidines 225C, 225H | Alkaloids |
| 18 | 7.3, 9.3 | 238.2163 | C_15_H_28_NO | 2.5 | -0.141 | - | PTX 237A, 3,5-Disubstituted Pyrrolizidine 237G R, 5,6,8-Trisubstituted indolizidine 237C L M N S, Histrionicotoxin 237F | Alkaloids |
| 19 | 13.50 | 240.1961 | C_14_H_26_O_2_N | 2.5 | 0.224 | 223.17 (100), 187.15 (32), 205.16 (12), 95.09 (11), 196.17 (8), 71.81 (7), 222.19 (7) | hPTX 239M | Alkaloids |
| 20 | 9.35 | 240.2320 | C_15_H_30_ON | 1.5 | 0.151 | 108.96 (100), 183.13 (83), 94.81 (74), 223.08 (63), 198.69 (40), 212.1 (35), 222.07 (33), 122.99 (19) | 3,5-Disubstituted indolizidines 239, 5,6,8-Trisubstituted indolizidines 239W, histrionicotoxin 239H | Alkaloids |
| 21 | 9.18 | 242.2476 | C_15_H_32_NO | 0.5 | -0.371 | - | 4-Hydroxy-2,6-Disubstituted Piperidine 241D | Alkaloids |
| 22 | 9,63 | 252.2323 | C_16_H_30_ON | 2.5 | 0.159 |  | Pumiliotoxin 251D | Alkaloids |
| 23 | 13.11 | 254.21132 | C_15_H_28_NO_2_ | 2.5 | -0.046 | - | PTX 253F, aPTX 253A, Decahydroquinoline 253D | Alkaloids |
| 24 | 8.92 - 12 | 266.2476 | C_17_H_32_ON | 2.5 | 0.019 | - | Cyclopentaquinolizidine 265 B, 8-Deoxypumiliotoxin 265X, 8-Desmethylpumiliotoxin 265V, Homopumiliotoxin 265N, Histrionicotoxin 265E | Alkaloids |
| 25 | 9.54 | 268.2271 | C_16_H_30_O_2_N | 2.5 | 0.004 | 250.22 (100), 196.13 (2) | Homopumiliotoxin 267P, Allopumiliotoxin 267A, Pumiliotoxin 267C, 9-Desmethyl-Homopumiliotoxin 267N | Alkaloids |
| 26 | 14.8 | 268.2634 | C_17_H_34_ON | 1.5 | 0.061 | - | 5,6,8-Trisubstituted indolizidines 267 R | Alkaloids |
| 27 | 12.45 | 274.2528 | C_19_H_32_N | 4.5 | -0.027 | - | 5,8-Disubstituted Indolizidine 273B C, 5,6,8-Trisubstituted indolizidine 273A | Alkaloids |
| 28 | 9.61 | 276.2321 | C_18_H_30_ON | 4.5 | 0.289 | - | PTX 275H | Alkaloids |
| 29 | 16.4 | 278.21138 | C_17_H_28_NO_2_ | 4.5 | 0.274 | - | PTX 277B | Alkaloids |
| 30 | 8.35, 14.71 | 278.24779 | C_18_H_32_NO | 3.5 | -0.531 | - | PTX 277G | Alkaloids |
| 31 | 15.56, 18 | 280.2634 | C_18_H_34_NO | 2.5 | 0.029 | 263.24 (100), 245.23 (57), 265.02 (18), 287.05 (11), 196.79 (8) | Cyclopentaquinolizidine 279B C | Alkaloids |
| 32 | 7, 9.76 | 282.2426 | C_17_H_32_NO_2_ | 2.5 | -0.236 | - | 8-Deoxy-PTX 281B N, hPTX 281K | Alkaloids |
| 33 | 9.28 | 284.2007 | C_17_H_32_NO_2_ | 7.5 | 0.009 | - | Histrionicotoxin 283A | Alkaloids |
| 34 | 8.85 | 286.2159 | C_19_H_28_NO | 6.5 | -0.841 | - | Histrionicotoxin 286A B C E | Alkaloids |
| 35 | 11.7 | 294.2426 | C_18_H_32_NO_2_ | 3.5 | 0.254 | - | PTX 293E | Alkaloids |
| 36 | 19 | 294.2790 | C_19_H_36_NO | 2.5 | 0.309 | 287.31 (100), 196.11 (74), 71.83 (37), 265.25 (34), 167.15 (31), 196.21 (30) | 8-Deoxypumiliotoxin 293D | Alkaloids |
| 37 | 16 | 296.2583 | C_18_H_34_NO_2_ | 2.5 | 0.074 | - | 8-Deoxypumiliotoxin 295C | Alkaloids |
| 38 | 11.89 | 312.2532 | C_18_H_34_NO_3_ | 2.5 | -0.120 | - | Allopumiliotoxin 311 | Alkaloids |
| 39 | 7.9 | 326.2685 | C_19_H_36_NO_3_ | 2.5 | -0.350 | - | PTX325B, aPTX 325A | Alkaloids |
| 40 | 6, 8.23 | 338.2687 | C_20_H_36_NO_3_ | 3.5 | 0.040 | - | hPTX 337A B | Alkaloids |

^1^Tentative annotation was based on (Daly *et al*., 2005)

**S2 Table.** Ant fauna of Karpathos, Kasos and Kastellorizo islands.

|  | **Ant subfamilies** | | | **Ant genera** | | | |
| --- | --- | --- | --- | --- | --- | --- | --- |
| **ISLAND** | |  | **Karpathos** | | **Kasos** | **Kastellorizo** | |
|  | | Ponerinae | *Cryptopone* | |  | |  |
|  | |  | *Hypoponera* | |  | |  |
|  | | Myrmicinae | *Aphaenogaster* | | *Messor* | |  |
|  | |  | *Crematogaster* | | *Monomorium* | |  |
|  | |  | *Temnothorax* | | *Pheidole* | |  |
|  | |  | *Messor* | | *Tetramorium* | |  |
|  | |  | *Monomorium* | |  | |  |
|  | |  | *Oxyopomyrmex* | |  | |  |
|  | |  | *Pheidole* | |  | |  |
|  | |  | *Solenopsis* | |  | |  |
|  | |  | *Strongylognathus* | |  | |  |
|  | |  | *Temnothorax* | |  | |  |
|  | |  | *Tetramorium* | |  | |  |
|  | | Dolichonerinae | *Tapinoma* | | *Tapinoma* | |  |
|  | | Formicinae | *Lepisiota* | | *Lepisiota* | | *Camponotus* |
|  | |  | *Acropyga* | | *Camponotus* | |  |
|  | |  | *Camponotus* | | *Plagiolepis* | |  |
|  | |  | *Lasius* | |  | |  |
|  | |  | *Nylanderia* | |  | |  |
|  | |  | *Plagiolepis* | |  | |  |

| **Mite Order** | **Prostigmata** | **Oribatida** | | |
| --- | --- | --- | --- | --- |
| **Mite Family** | **Eriophyidae** | **Aphelacaridae** | **Brachychthoniidae** | **Liodidae** |
| Mite *Species* | *Eriophyes oleae* | *Aphelacarus acarinus* | *Brachychochthonius hauserorum* | *Platyliodes doderleinii* |
|  |  |  | *Brachychochthonius hungaricus* |  |
|  |  |  | *Brachychthonius hauserorum* |  |
|  |  |  | *Brachychthonius hungaricus* |  |
|  |  |  | *Brachychthonius immaculatus* |  |
|  |  |  | *Liochthonius brevis* |  |
|  |  |  | *Liochthonius strenzkei* |  |

**S3 Table.** Mite fauna of Karpathos, Kasos and Kastellorizo islands.

**S4 Table.** Diagnostic compounds of Ag. Mammas population or Kasos Island

| **AA** | **Retention time (min)** | **Detected** ***m/z***  **([M+H]^+^)** | **MS/MS *m/z***  **(relative intensity)** | **Proposed formula** | **RDBeq.** | ***Δ* (ppm)** | **Proposed compounds** | **Chemical group** |
| --- | --- | --- | --- | --- | --- | --- | --- | --- |
| 1 | 8.3 | 331.1648 | - | C_18_H_23_N_2_O_4_ | 8.5 | -0.444 |  | Alkaloids |
| 2 | 6.45 | 334.1647 | 316.30 (100), 255.22 (66.17), 273.26 (31.96), 334.33 (23.46), 317.30 (19.17), 273.29 (19.11), 290.24 (13.15), 133.04 (11.55), 159.05 (10.75) | C_18_H_24_O_5_N | 7.5 | 0.601 | Seneciphylline | Alkaloids |
| 3 | 7 | 335.1686 |  |  |  |  |  |  |
| 4 | 7.8 | 336.1802 | 308.19 (100), 336.29 (71.67), 120.08 (29.52), 335.17 (28.36), 336.29 (28.23), 138.09 (18.89) | C_18_H_26_O_5_N | 6.5 | 0.141 | Senecionine, | Alkaloids |
| 5 | 7.8 | 337.1836 |  |  |  |  |  |  |
| 6 | 8.16 | 366.1907 | 348.18 (100), 338.20 (54.42), 366.19 (50.67), 348.22 (27.96), 351.14 (19.01), 356.49 (16.08) | C_19_H_28_O_6_N | 6.5 | 0.5 | Senkirkine | Alkaloids |
| 7 | 8.68 | 370.1647 | 339.12 (100), 313.11 (51.23), 327.12 (46.07), 370.17 (28.46), 338.14 (27.37) | C_21_H_24_NO_5_ | 10.5 | -0.154 | - | Alkaloids |
